# Designing a multi-epitope vaccine against *Mycobacteroides abscessus* by pangenome-reverse vaccinology

**DOI:** 10.1038/s41598-021-90868-2

**Published:** 2021-05-27

**Authors:** Hamza Arshad Dar, Saba Ismail, Yasir Waheed, Sajjad Ahmad, Zubia Jamil, Hafsa Aziz, Helal F. Hetta, Khalid Muhammad

**Affiliations:** 1 Foundation University Medical College, Foundation University Islamabad, DHA-I, Islamabad, 44000 Pakistan; 2 Nuclear Medicine, Oncology, and Radiotherapy Institute, Islamabad, 44000 Pakistan; 3 Department of Medical Microbiology and Immunology, Faculty of Medicine, Assiut University, Assiut, 71515 Egypt; 4 Department of Biology, College of Science, United Arab Emirates University, 15551 Al Ain, United Arab Emirates

**Keywords:** Computational biology and bioinformatics, Immunology, Microbiology

## Abstract

*Mycobacteroides abscessus* (Previously *Mycobacterium abscessus*) is an emerging microorganism of the newly defined genera Mycobacteroides that causes mainly skin and tissue diseases in humans. The recent availability of total 34 fully sequenced genomes of different strains belonging to this species has provided an opportunity to utilize this genomics data to gain novel insights and guide the development of specific antimicrobial therapies. In the present study, we collected collectively 34 complete genome sequences of *M. abscessus* from the NCBI GenBank database. Pangenome analysis was conducted on these genomes to understand the genetic diversity and to obtain proteins associated with its core genome. These core proteins were then subjected to various subtractive filters to identify potential antigenic targets that were subjected to multi-epitope vaccine design. Our analysis projected the open pangenome of *M. abscessus* containing 3443 core genes. After applying various stepwise filtration steps on the core proteins, a total of four potential antigenic targets were identified. Utilizing their constituent CD4 and CD8 T-cell epitopes, a multi-epitope based subunit vaccine was computationally designed. Sequence-based analysis as well as structural characterization revealed the immunological effectiveness of this designed vaccine. Further molecular docking, molecular dynamics simulation and binding free energy estimation with Toll-like receptor 2 indicated strong structural associations of the vaccine with the immune receptor. The promising results are encouraging and need to be validated by additional wet laboratory studies for confirmation.

## Introduction

*Mycobacteroides abscessus* (Previously called *Mycobacterium abscessus*) is an emerging microorganism that is increasingly becoming resistant to antibiotic therapy and causes diseases related to skin and soft tissues^1,2^. According to the revised bacterial nomenclature system, Mycobacteroides is the name of new genera (proposed in 2018) that was identified on the basis of 51 unique molecular markers and belongs to the Abscessus-Chelonae clade that generally exhibits fast growth^3,4^.

*Mycobacteroides abscessus* is a highly notorious and common member of the Mycobacteroides genus. *M. abscessus* commonly poses numerous therapeutic challenges on account of its innate resistance to majorly used antibiotics^5^. Some mortal cases in Cystic Fibrosis patients have also been reportedly linked to these infections^6^. The seriousness of this situation can be gauged by the fact that in many countries, lung transplantation is not recommended in case of *M. abscessus* infection thus rendering CF patients hopeless. The pathology of their infection is mostly related to a transition from a smooth “S” form to rough the “R” form^7^. The emergence of this bacterial species has been largely attributed to the increased use of broad-spectrum antibiotic therapy.

The introduction of Next Generation Sequencing and other whole genome sequencing-based technologies has revolutionized the field of genomics^8^. This, along with recent advancements in the field of bioinformatics, has resulted in the accumulation of substantial genomics data of many microorganisms in publicly available biological databases. At the time of writing, a total of 34 genome sequences of *M. abscessus* were available in the GenBank. Therefore, we aimed to explore this data using bioinformatics tools to enhance our understanding of the genome scale analysis of this opportunistic microorganism and identify potential targets to guide vaccine discovery. This was important considering that conventional approaches in vaccinology take a lot of time, resources and expertise^9^. Briefly, these studies involve complicated steps like microbial culturing, isolation of antigen, inactivation and reinjection of these antigens into subjects to verify the immunogenic potential. Furthermore, these classical approaches are not feasible to conduct large scale analysis on a variety of bacterial isolates/strains. In such situations, there is a need to identify conserved antigenic target, prepare it, and evaluate its utility for immunization applications.

This study attempted to address the therapeutic challenges arising due to the worsening antimicrobial resistance within *M. abscessus*. For this purpose, complete genome sequences of this opportunistic bacterial pathogen were obtained from the NCBI GenBank database. An integrative analysis was thus conducted encompassing pangenome analysis, reverse vaccinology, immunoinformatics as well as structural bioinformatics to identify promising targets to design a universally applicable chimeric vaccine. This was especially relevant considering the lack of such studies in the literature concerning an emerging microorganism of public health interest.

We believe that the putative vaccine targets fulfilled the criteria to act as a source of epitopes for developing a polyepitope vaccine. Further the three-dimensional structure prediction was performed on the multi-epitope vaccine. Molecular docking analysis with Toll-like receptor 2 was also conducted to evaluate the vaccine's effectiveness. Altogether, we believe that this study will provide updated knowledge about the global gene repertoire of an emerging bacterial species and guide novel vaccine design to counter these infections.

## Material and methods

### Collection of genomics data

The genomics and proteomics data of total 34 complete *M. abscessus* genomes was collected from the GenBank database of NCBI. Additional information, such as strain name, GC%, accession number, and number of contigs, was also collected.

### Pangenome analysis

Pangenome analysis was carried out on the 34 genomes using the BPGA, a quick genome analysis pipeline^10^. For clustering sequences, a 90% sequence identity threshold was specified in USEARCH algorithm^11^. BPGA standalone tool computes pan and core genome size by 20 permutations and stating median values after the inclusion of every genome. The core and pan genome graphs are obtained by analyzing the total number of common and unique gene families against the total number of genomes, respectively. Additionally, BPGA can generate a gene family distribution plots illustrating core pan and accessory genes. The output also yields a distribution plot of new genes which shows how many new genes are added with the inclusion of each successive genome. The protein sequences associated with the *M. abscessus* core genome were also obtained and subjected to additional filters to identify potential therapeutic targets.

### Reverse vaccinology approach to select Potential Vaccine Candidates

To guide the selection of potential core vaccine targets, Vaxign server (http://www.violinet.org/vaxign/) was used. Vaxign is the first reverse vaccinology tool to identify suitable antigenic targets in bacterial genomes (mostly)^12^. For this purpose, “Dynamic Analysis” mode was used to query the core genome-related proteins.

The first inclusion criteria applied to the core genome proteins were subcellular localization restricted to the cell wall or extracellular regions. Exoproteome and secreted substances associated with bacteria are considered to be useful antigenically as they are exposed outside the bacterial cell and can easily develop interactions with circulating immune cells^13^. For cross-validation of subcellular localization, two popular bioinformatics servers were used: PSORTb v3.0.2 (available at http://www.psort.org/psortb/) and CELLO v.2.5 (available at http://cello.life.nctu.edu.tw/).

The exclusion criteria applied to the non-homologous proteins was homology to humans and related organisms (mice and pig). Due to significant similarity with the human proteins, either autoimmunity or tolerance to foreign antigens could occur^14^. This can have harmful consequences for human health. Therefore, bacterial proteins found to exhibit homology to host protein(s) are generally not good candidates for developing a safe vaccine.

The adhesion potential of targets was assessed using Vaxign. The threshold for adhesion selection was set to 0.5. Proteins having adhesive properties interact with the host cellular receptors and facilitate bacterial attachment^15^. Specific antibodies can thus be generated against these foreign proteins by the host and may serve to activate protective immunity^16^. Thus, we took an aim to explore this aspect.

The final prioritization step by Vaxign was the number of transmembrane helices less than two. This is usually suggested to ensure that purification of these targets is done in an appropriate wet laboratory setting^9^.

Next, the antigenic tendency of the predicted proteins was analyzed using the VaxiJen 2.0 server^17^. Score cut-off was adjusted to 0.5. The good thing about this server is the lack of dependence on alignments. With accuracy within 70–89% range, this tool is known to be very accurate and may lead to fewer false positive hits, which is highly desirable in the reverse vaccinology approach.

The Protparam tool was used to compute the physicochemical features of selected proteins^18^. In particular, four things were assessed. Firstly, molecular weights were analyzed to verify whether they are of low weight (less than 110 kD)^19^. Secondly, the Instability Index was checked to confirm stability. Finally, the hydrophobicity and aliphatic index were scrutinized to select hydrophilic molecules with good thermal stability. The negative GRAVY index provided an indication of hydrophilicity whereas a higher aliphatic index (> 50) suggested high stability at varying temperatures^20^.

### Prediction of CD8 T cell interacting epitopes

HLA class I epitope prediction was done through VaxiTop analysis through Vaxign server^12^. Default *P* value (0.05) was selected, the alleles specified were HLA-A0101, HLA-A0201, HLA-A0301, HLA-A1101 and HLA-B3501, organism selected was human, and only epitopes lying outside cell were considered due to their maximum exposure to the immune cells outside the cell. The predicted epitopes were further scrutinized on the basis of their immunogenicity scores calculated by the immunogenicity tool of the IEDB^21,22^. The antigenicity filter was also applied on the proteins by the VaxiJen 2.0 server, with 0.8 cut-off score^17^. Finally, the allergenicity of the 9-mer peptide sequences was evaluated using the AllergenFP 1.0 tool to remove potential allergens^23^.

### Prediction of CD4 T cell interacting epitopes

To predict T-cell epitopes having the tendency to interact with HLA class II, NetMHCIIpan 3.2 server (available at http://www.cbs.dtu.dk/services/NetMHCIIpan-3.2/) was used (Jensen et al., 2018). Default threshold for categorizing binding affinity level to shortlist strong binders. DRB alleles specified were DRB1_0101, DRB1_0701, DRB1_0301, DRB1_0401, DRB1_0801, DRB1_1101, DRB1_1501 and DRB1_1301. The predicted results were sorted as per the binding affinity values. The antigenicity screening of top binders for each allele were conducted using the VaxiJen server^17^. Only those epitopes scoring above 0.5 threshold were selected. Wherever there were overlapping peptides, those having the most antigenic values were retained for that allele.

### In silico design of multi-epitope vaccine and structural modeling

The shortlisted epitopes were attached to each other using a specialized Glycine-Proline linker sequence of GPGPG. Towards the N-terminal direction of the epitopes, Cholera Toxin subunit B, a molecular adjuvant was incorporated. This adjuvant was connected to the epitopes via one EAAAK rigid linker. The physical and chemical properties of vaccine such as theoretical pH, half-life, molecular weight, grand average of hydropathicity, aliphatic and instability indexes, were computationally analyzed using the Expasy tool of Protparam^18^. Furthermore, the designed multi-epitope vaccine was subjected to further antigenicity and allergenicity checks, using VaxiJen and AllergenFP servers, respectively^17,23^.

After sequence-based analysis, the three-dimensional structure prediction was performed. For this purpose, the 3DPro tool was utilized^24^. The crude model obtained was then subjected to further improvements via Galaxyrefine 2 server through simulations to enhance its structural quality^25^. This was corroborated through different tools like ProSA-web and PROCHECK to ensure the availability of a structurally refined and stable multi-epitope vaccine for the next steps^26,27^.

### In silico C-immune simulation

The designed construct was subjected to C-ImmSim server for determination of its in silico immunogenic potential^28^. The server operates by application of machine learning techniques and position-specific scoring matrix (PSSM) for evaluation of host immune response towards the antigen.

### Vaccine-TLR molecular docking analysis

The binding affinity of the vaccine to TLR2 was explored using a molecular docking technique to validate the vaccine's immunological effectiveness. *M. abscessus* infection leads to TLR2-mediated immune responses in humans^29^. The structure of TLR2 was obtained from a crystal structure with PDB ID 2Z7X ^30^.

The protein structural files PDB were uploaded to the HADDOCK system guru interface using default options^31^. To obtain active and passive residues, the CPORT server was used^32^. Docking clusters were obtained and one cluster with the minimum HADDOCK score was uncovered. From this cluster, one docked PDB file was proceeded towards the refinement stage to improve the orientation of biomolecules towards one another. The UCSF Chimera tool^33^ and VMD^34^ visualized the docked vaccine-TLR structure. To identify and highlight the crucial binding interface within the docked complex, PDBsum server was used^35^.

### Binding affinity analysis of vaccine-TLR2 complex

The vaccine-TLR2 complex was submitted to the PRODIGY server (available at http://milou.science.uu.nl/services/PRODIGY) to predict the vaccine-TLR2 binding strength^36^. The prediction is carried out by exploiting information such as intermolecular contacts as well as non-interface properties. Default parameters were selected to conduct this analysis.

### Molecular dynamics simulation

The docked complex of vaccine-TLR2 was subjected to a 50 ns production run of molecular dynamics simulation to understand the system dynamics in aqueous solution. Molecular dynamics simulation was performed using AMBER18 software^37^. Both TLR2 and vaccine parameters were prepared using the ff14SB force field^38^. Complex integration into a TIP3P water box was accomplished in the process where the padding distance was set to 12 Å. Neutralization of the system was done by adding Na+ ions. System hydrogen atoms, solvation box, carbon alpha atoms, and all non-heavy atoms were minimized for 500, 1000, 1000, and 300 steps, respectively. Subsequently, heating of the system to 300 K for 20-ps was done via Langevin dynamics to maintain the system temperature^39^. Here, the restraint of 5 kcal/mol-A2 on carbon alpha atoms at time step of 2 fs was allowed. In equilibration, system was relaxed for 100 ps. System pressure was maintained by means of NPT ensemble for 50-ps. Lastly, a production run of 50 ns was accomplished at the time interval of 2 fs. Generated trajectories were analyzed for structural parameters using AMBER CPPTRAJ^40^. The hydrogen bonds formed throughout the trajectories between TLR2 and vaccine were plotted in VMD^34^, setting an angle of 30° and a bond distance of 0.35 nm.

### MM-PB/GBSA studies

Intermolecular binding free energies of the system were estimated using MMPBSA.py package of AMBER18 program^41^. The overall objective of this analysis was to evaluate the free energy difference between two states (solvated and gas phase) of the complex. The net binding free energy was calculated as,

1 $$\begin{aligned} \Delta G \;binding\;free\;energy & = \Delta G\;bind, \;vaccum + \Delta G\;solv, \;complex \\ & \quad {-}\left( {\Delta G \;solv, \;ligand} \right) + \Delta G\;solv, \;receptor \\ \end{aligned}$$

2 $$\begin{aligned} \Delta G\; solv & = \Delta G\;electrostatic,\varepsilon = 80 + \Delta G \;electrostatic,\varepsilon \\ & = 1 + \Delta G \;hydrophobic \\ \end{aligned}$$

3 $$\Delta G\;vaccum = \Delta E\;molecular, \;mechanics {-} T \cdot \Delta G\; normal\;\bmod e\;analysis$$

In total, 100 frames were extracted from simulation trajectories to be used in both MMGBSA (Molecular Mechanics Generalized Born Surface Area) and MMPBSA (Molecular Mechanics Poisson–Boltzmann Surface Area).

## Results

### Pangenome analysis and retrieval of the core proteome

**Figure 1:**
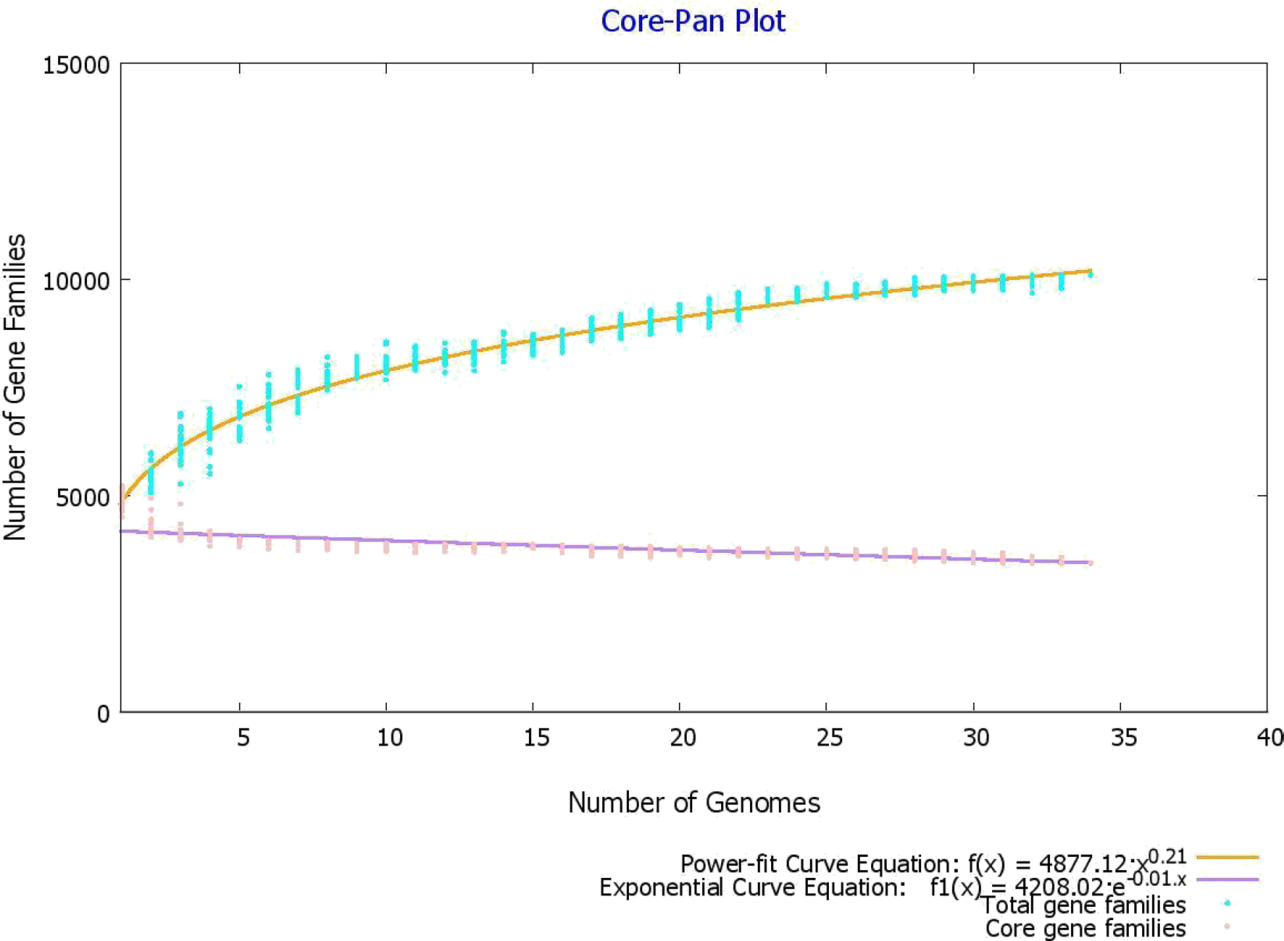
Pan-core plot of 34 *Mycobacteroides abscessus* genomes. With the inclusion of each new genome, the size of the pangenome increased while that of the core genome decreased. The pangenome curve (shown in brown) continues to show an upward trajectory. This suggests that the global gene pool of *M. abscessus* is likely to increase in the future.

**Figure 2:**
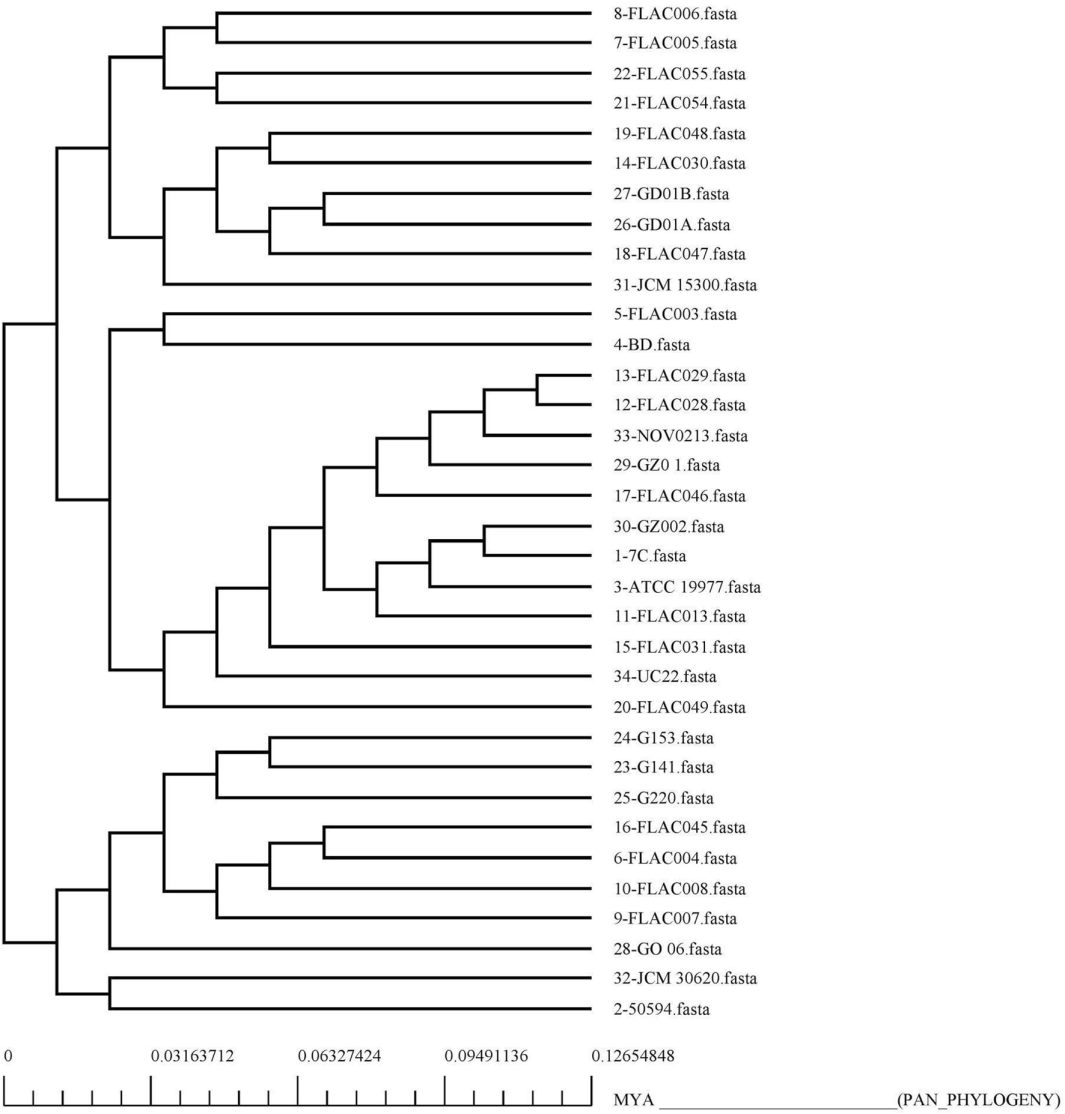
Panphylogeny tree of 34 complete genomes of *Mycobacteroides abscessus*. This pan-phylogeny has been constructed on the basis of accessory gene presence/absence data in different strains.

**Figure 3:**
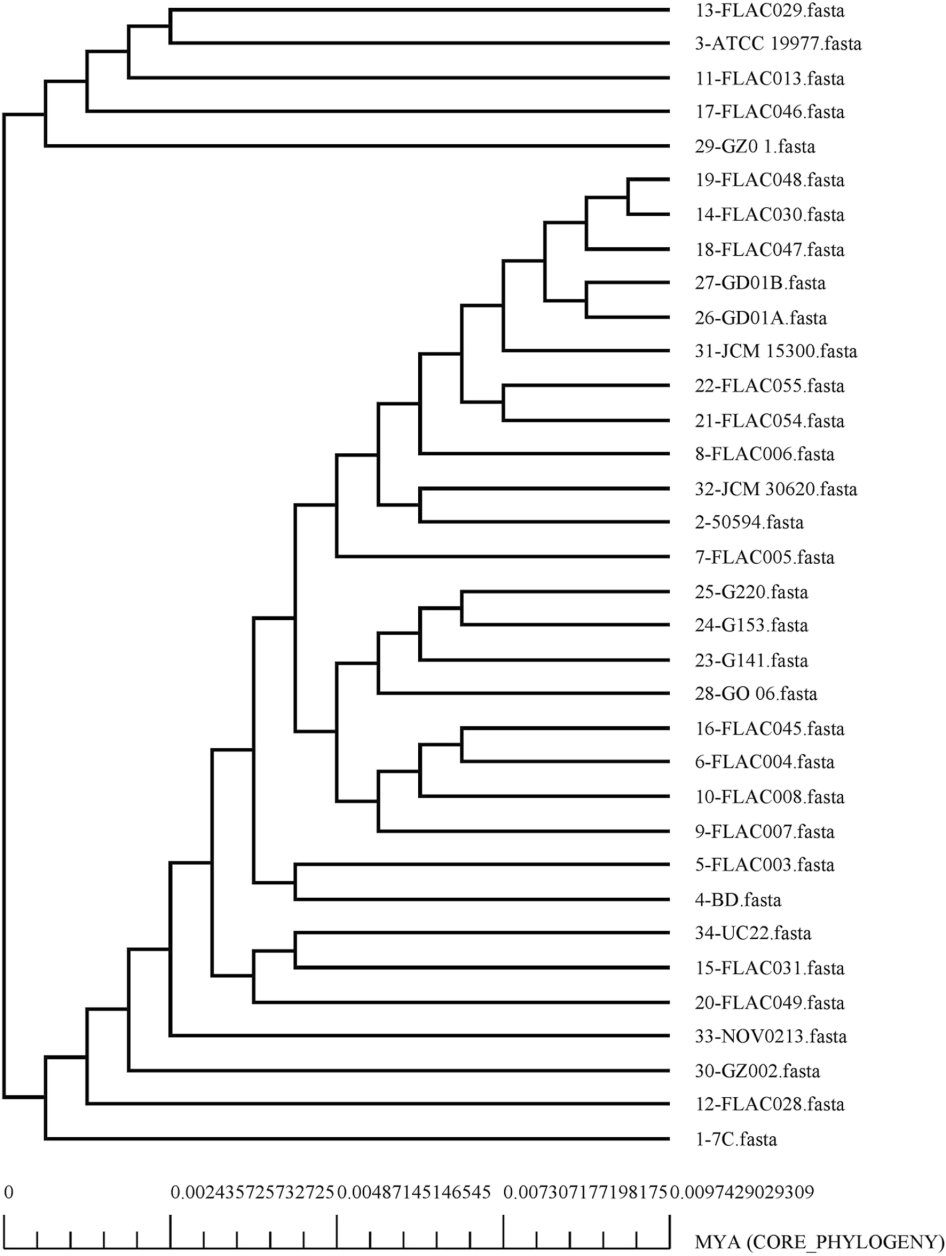
Core genome tree of 34 complete genomes of *Mycobacteroides abscessus*. This core phylogeny has been obtained on the basis of concatenated alignment of proteins associated with the core genome.

34 complete genomes of *M. abscessus* were obtained from GenBank. The information of these strains such as strain name, accession number, as well as genome statistics are provided in Supplementary file 1. It was found through pangenome analysis of *M. abscessus* strains that its core genome size is 3443. Meanwhile, the total number of gene families (pangenome) was calculated to be 10,110. The core/pangenome size ratio was found to be 0.340, thus the core forms 34% of the pangenome. This signifies the high level of genetic diversity in the *M. abscessus* species. The pan-core plot (Fig. 1) also visualized this trend. The expansion rate 'b' was found to be 0.2093 which indicates that the pangenome of this species is open and has the potential to increase. Each genome on average contained a total of 4893 protein-encoding genes. The core genome size accounted on average for 70% of average genome size. The smallest number of protein-encoding genes were present in GO 06 strain i.e., 45,212; Meanwhile, strain FLAC055 contained a total of 5237 genes. The pangenome and core genome phylogenetic trees were constructed and subsequently visualized (Figs. 2 and 3).

### Identification of potential core vaccine candidates in *Mycobacteroides abscessus*

The analysis of the core genome-associated proteins through Vaxign revealed that out of 4443 proteins, only 45 had subcellular localization restricted/confined to the extracellular or cell wall regions (Supplementary file 2).

The homology analysis of these confirmed that the total six targets were homologs of humans, mice and/or pigs, hence they were removed from further consideration to retain 39 proteins (Supplementary file 3). Next, the adhesion filter was applied, and it was found that a total of 19 proteins had putative adhesion properties (Supplementary file 4). All of them had less than two transmembrane helices.

After Vaxign, additional filters were applied. The antigenicity evaluation of the targets revealed that the total 10 targets had antigenic tendencies and thus were good vaccine candidates (Supplementary file 5). It was found through physicochemical analysis that out of these 10 proteins, only four exhibited favorable properties such as low molecular weight, high aliphatic index and instability index less than 40 as well as negative value of the GRAVY index (Supplementary file 6). These observations further support the immunological relevance of our polyepitope vaccine.

Subcellular localization cross-check was performed on the 45 proteins predicted as extracellular or cell wall-localized by Vaxign for validation. PSORTb v3.0.2 revealed that 37 proteins were extracellular while the remaining eight were cell wall-localized targets (Supplementary file 7). Whereas, according to CELLO server, 24 out of 45 proteins were classified as extracellular and the remaining 21 were regarded as cytoplasmic/membrane or multiple-localized targets (Supplementary file 7). However, even by retaining this strict cross-verification standard, further prioritization steps of 24 extracellular proteins yielded the same four antigen targets obtained in our study. Also, all four antigens were classified as extracellular by both PSORTb v3.0.2 and CELLO v 2.5. So, as a whole, this analysis provided verification of subcellular localization through multiple credible tools.

BLAST analysis of these proteins revealed that two out of these were hypothetical proteins so their functional annotation is not available. Whereas the remaining two are Resuscitation-promoting factor (RPF) and invasion protein Inv1. RPF is present in numerous gram positive bacteria responsible for reactivating cultures from stationary phase. RPF family is very likely to act as trans glycosylase enzymes similar to PF00062 and PF01464 (http://academic.oup.com/femspd/article/58/1/39/557188). This protein also shares structural elements with lysozyme and associated proteins. Inv exact function is not known however it bears similarity to peptidoglycan endopeptidase RipA of *Mycobacterium tuberculosis* variant bovis BCG and cleaves the bond between D-glutamate and meso-diaminopimelate according to Uniprot (https://www.uniprot.org/).

### Identification of potential HLA class I epitopes in PVCs

Analyzing the sequence of four core antigenic proteins using the VaxiTop resulted in the identification of 82 HLA I T-cell epitopes (Supplementary file 8). Out of these, a total of 44 epitopes were positively selected on account of their immunogenicity as reflected by the positive immunogenicity scores by the IEDB server (Supplementary file 9). Further scrutinization of antigenicity and non-allergenicity prioritized four HLA I T-cell epitopes. These are QADETNATM, RVAENVLAR, LTATNPDDV and YYGGLQFNL.

### Identification of potential HLA class II epitopes in PVCs

Analysis through NetMHCIIpan3.2 server revealed the presence of total 23 HLA class II epitopes within the four core antigenic proteins (Supplementary file 10). After antigenicity screening and removal of overlapping epitopes, a total of four epitopes were retained. These are LWAMAPALVAAPMAL, LLIFAFLGVTAAVGV, GGDLELFKNATATMK, and SREEQIRVAENVLAR.

### The in silico design of polyepitope vaccine and sequence-based evaluation

The prioritized epitopes were linked together and with Cholera Toxin B to envision a multi-epitope subunit vaccine. Since HLA I T-cell epitope RVAENVLAR was found to lie within the prioritized HLA II T-cell epitope SREEQIRVAENVLAR, so out of these two, only the later epitope was incorporated. The polyepitope vaccine is comprised of 226 number of amino acids. Molecular weight of the recombinant antigen was found to be 23,657.02 Daltons. Isoelectric pH value is 5.81. The half-life is 30 h (mammalian reticulocytes, in vitro), at least 20 h (yeast, in vivo) and at least 10 h (*Escherichia coli*, in vivo). Whereas, its instability index was found to be ~ 30. If the instability index value of a given protein is less than 40, the protein is deemed to be stable. A significantly high aliphatic index of 79.16 was computed which projects a considerable level of thermostability. Finally, the GRAVY value was calculated, and it was revealed that this designed vaccine has a negative value of GRAVY index (− 0.143). This means that the vaccine is predicted to have a hydrophilic character and thus likely to engage closely with water, a desirable attribute of vaccine. The sequence of the polyepitope vaccine is provided below in plain format:

MTPQNITDLCAEYHNTQIHTLNDKIFSYTESLAGKREMAIITFKNGATFQVEVPGSQHIDSQKKAIERMKDTLRIAYLTEAKVEKLCVWNNKTPHAIAAISMANEAAAKQADETNATMGPGPGLTATNPDDVGPGPGYYGGLQFNLGPGPGSREEQIRVAENVLARGPGPGGGDLELFKNATATMKGPGPGLWAMAPALVAAPMALGPGPGLLIFAFLGVTAAVGV.

Both AllergenFP and AllerTOP 2.0 servers projected the non-allergenicity of multi-epitope vaccine. According to the VaxiJen server results, the antigenicity of the vaccine is 0.7973 i.e. above the threshold of 0.5 and thus highly antigenic. After sequence-based characterization, we proceeded towards structural analysis of the vaccine.

### Structural modeling of polyepitope-based subunit vaccine

**Figure 4:**
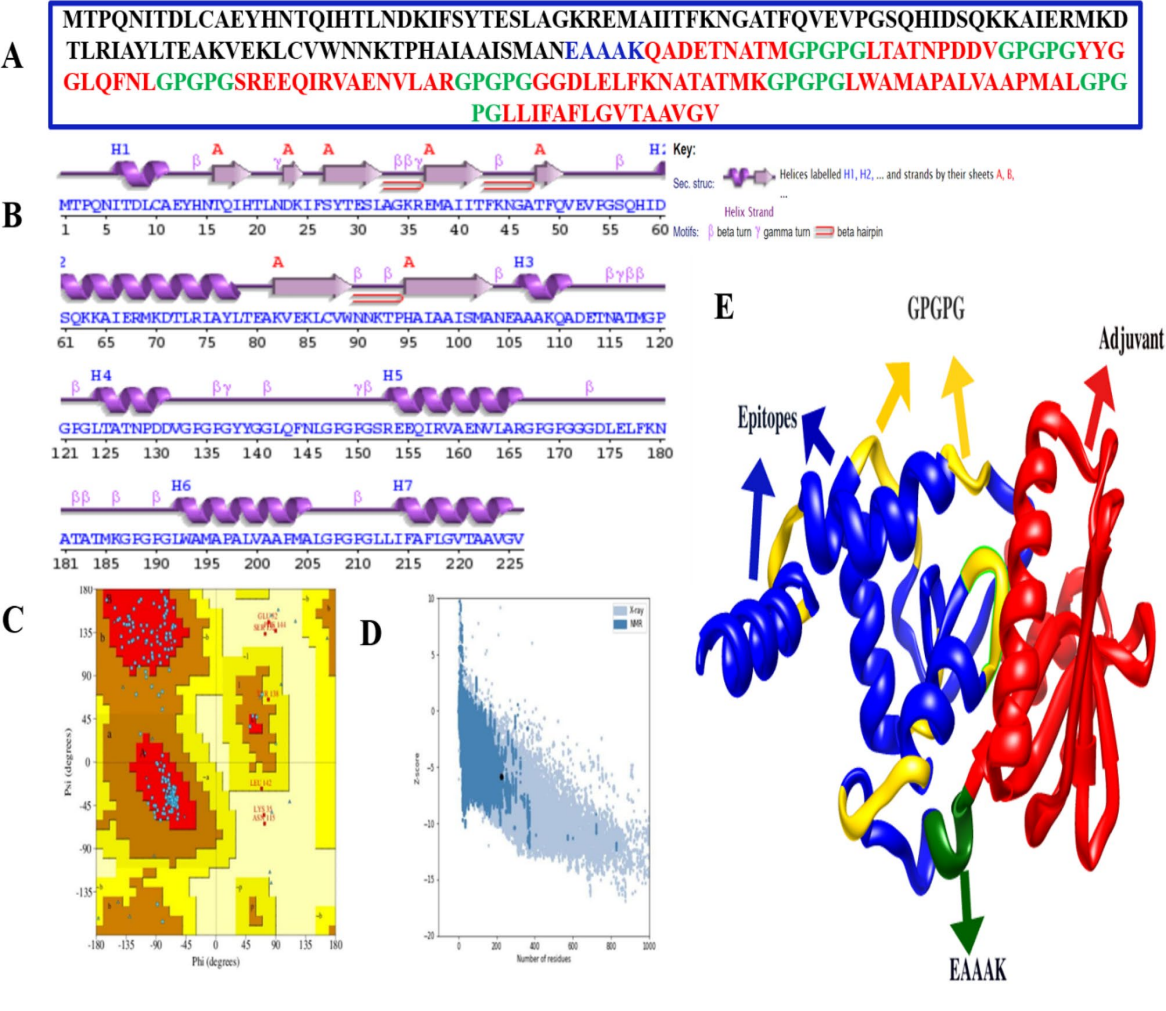
Structural characterization of multi-epitope vaccine construct: Polyepitope vaccine construct Sequence (**A**), Secondary Structure of polyepitope vaccine construct (**B**), PROCHECK graph of selected model (**C**), ProSA-web results showing Z-score of − 5.83(**D**), Cartoon visualization of the designed vaccine 3D structure (**E**).

Utilizing the intensive mode of the Phyre2 server, we were able to model a total of 142 residues (63%) with greater than 90% accuracy. However, critical assessment of this structure revealed that it was still in crude form and needed to be improved. Molecular refinements applied to this crude model yielded a total of ten models (Table 1). Out of these, the top three models according to GALAXY energy score were assessed further. Considering the Ramachandran plot analysis, ProSA-web and GALAXY energy results, MODEL1 was ultimately selected (Fig. 4).

**Table 1:** Information about structural properties of the ten models generated after refining the structure of the crude multi-epitope vaccine.

Model	RMSD	Mol probity	Clash score	Poor rotamers	Rama favored	GALAXY energy
Initial	0.000	4.145	180.6	6.5	73.2	52,633.20
MODEL 1	2.505	1.461	2.2	0.6	92.4	− 4411.11
MODEL 2	2.110	1.423	1.9	0.0	92.4	− 4398.08
MODEL 3	2.068	1.104	0.3	0.0	91.5	− 4396.93
MODEL 4	1.876	1.420	1.4	0.0	89.7	− 4379.94
MODEL 5	2.229	1.527	2.4	0.6	91.5	− 4369.58
MODEL 6	2.249	1.343	1.1	0.0	90.6	− 4368.67
MODEL 7	2.006	1.569	2.4	0.0	90.2	− 4364.43
MODEL 8	2.246	1.521	2.2	0.0	90.6	− 4361.25
MODEL 9	2.227	1.520	1.9	0.6	89.3	− 4359.12
MODEL 10	1.904	1.408	1.4	0.0	90.2	− 4350.07

### Conformational B-cell epitope mapping in designed subunit vaccine

**Figure 5:**
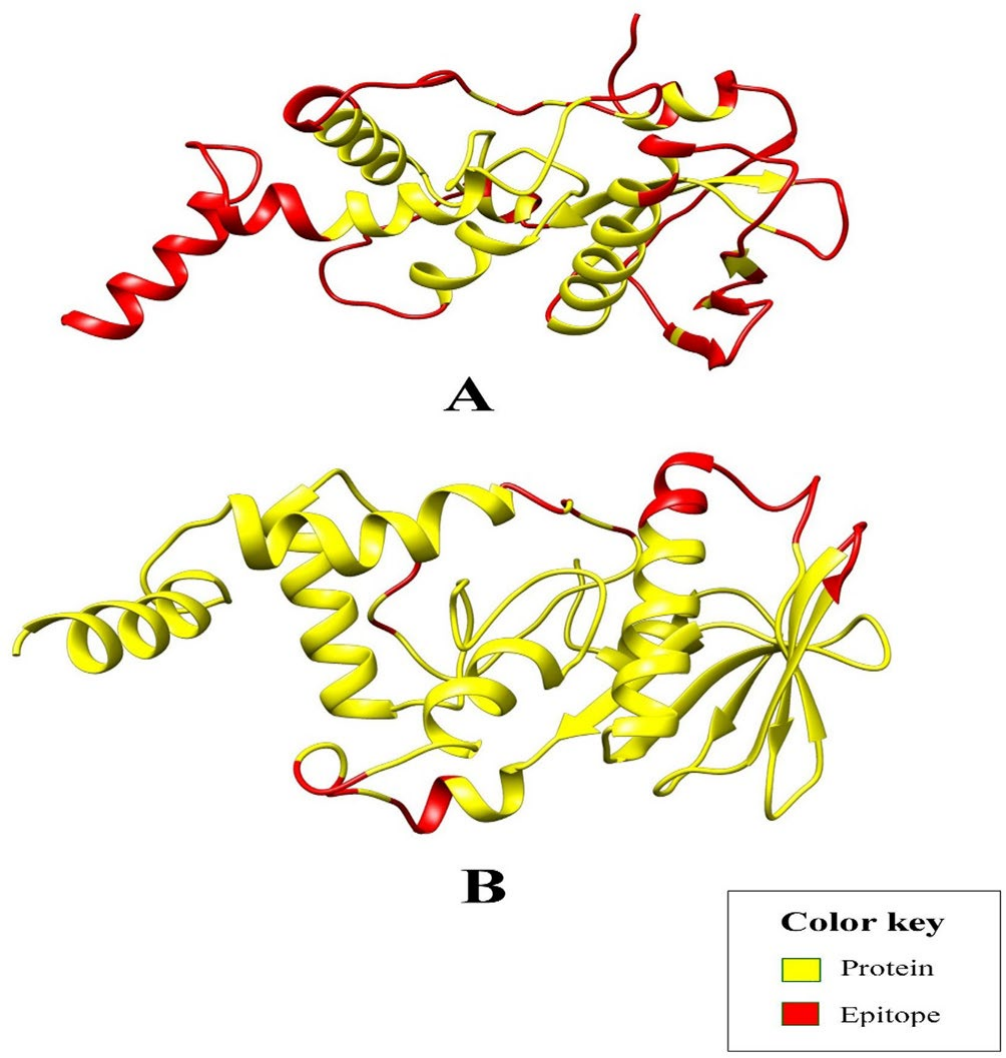
The discontinuous B-cell epitopes (red) within the multi-epitope vaccine as predicted by (**A**) Ellipro tool, and (**B**) DISCOTOPE server.

The three-dimensional structure of multi-epitope vaccine was subjected to discontinuous (conformational) B-cell epitopes prediction. A total of 126 epitopes were projected by the use of Ellipro server (Supplementary file 11). Whereas, DISCOTOPE server collectively predicted 33 B-cell epitopes (Supplementary file 12). Figure 5A shows the epitopes predicted by Ellipro, whereas Fig. 5B visualizes the epitopes predicted by DISCOTOPE.

### Population coverage of predicted T cell epitopes

**Figure 6:**
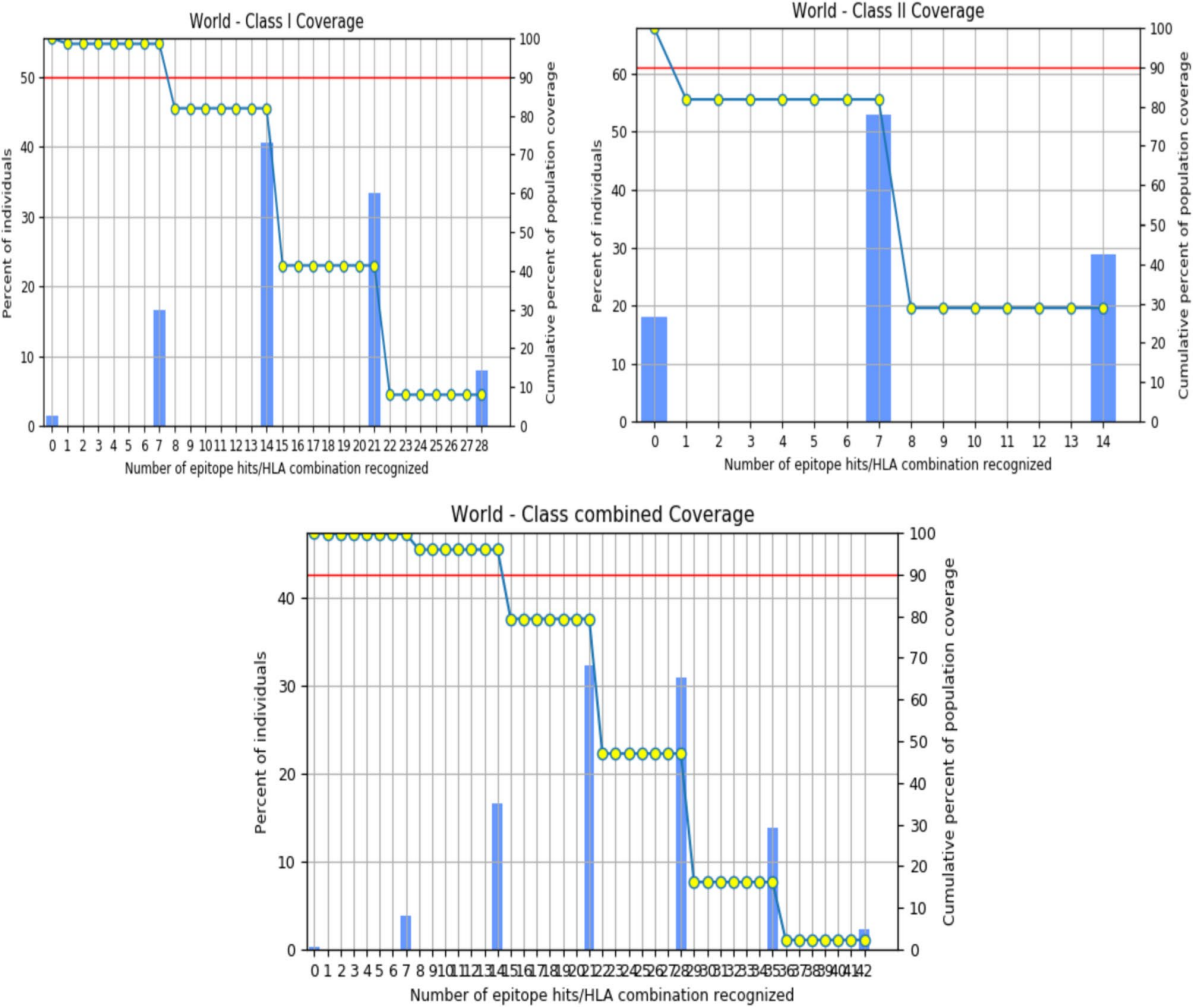
World class I, class II and combined coverage of HLA alleles recognized a set of predicted T cell epitopes.

In nature, MHC molecules are abundant and well distributed among various communities and in multiple ethnic groups. Therefore, the production of peptide-based vaccines, given most of the alleles, is a successful approach to broad-spectrum vaccine production. The T cell epitopes found in this study showed an average population coverage of 98.55% (MHC Class I), 81.81% (MHC Class II) and 99.74% (Combined). Figure 6 shows the known class I, class II and combined HLA alleles and the percentage of epitope group coverage.

### In silico cloning of polyepitope based vaccine construct

**Figure 7:**
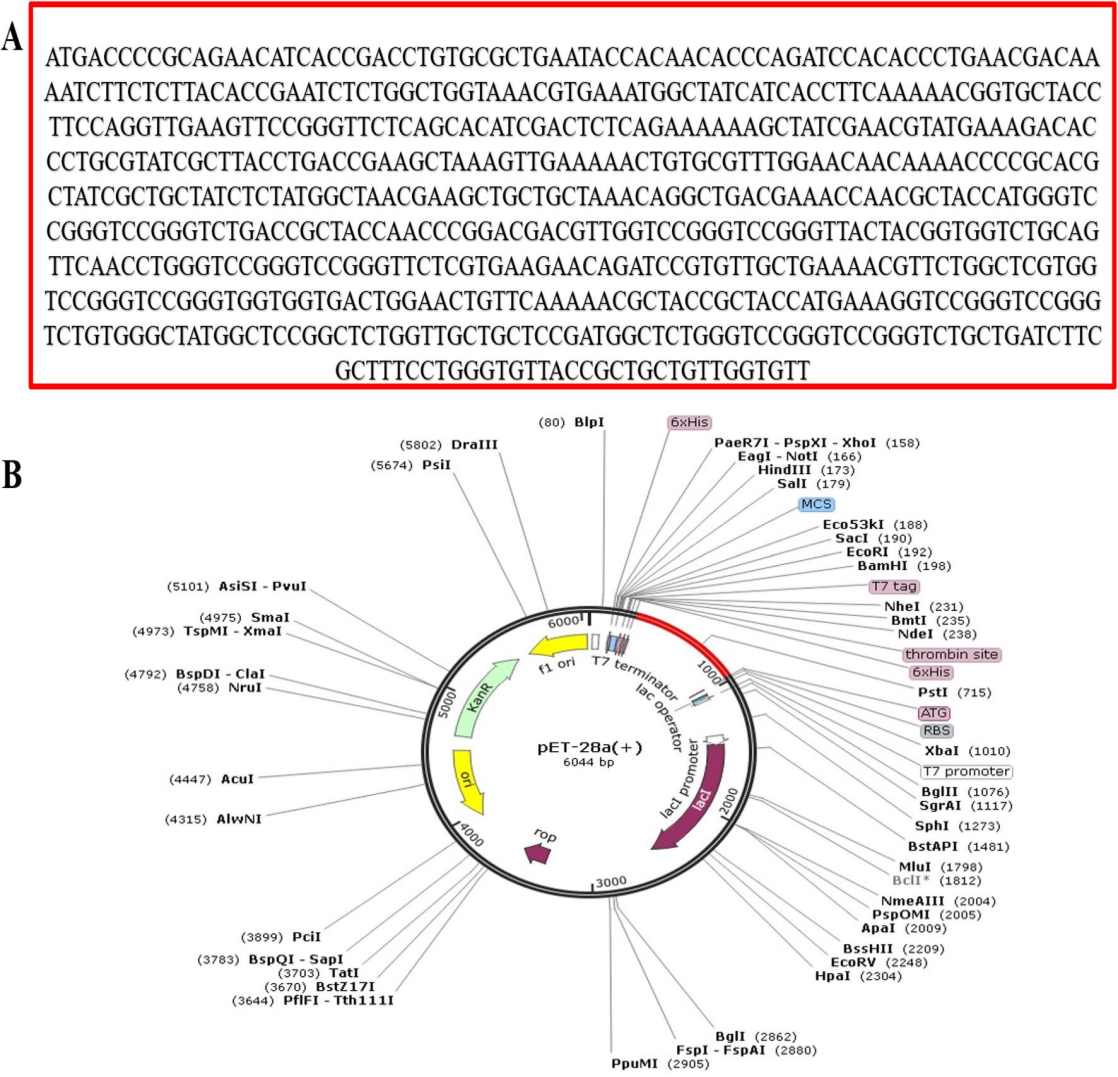
(**A**) The codon-optimized sequence of multi-epitope vaccine. (**B**) In silico cloning of polyepitope vaccine construct (shown in red) in pET28a expression vector.

The basic aim of in silico cloning of designed polyepitope based vaccine construct was to lead molecular biologists and genetic engineers on potential cloning sites and predict the level of expression in a particular expression system, for example in current study we used *E. coli* (K12) system. To produce its maximum expression, reverse translation of the designed polyepitope based vaccine construct’s primary sequence was performed followed by cloning (Fig. 7). The CAI value of the enhanced construct sequence is 1, suggesting the vaccine's ideal expression. Whereas the GC content is 50.6% almost to the *E. Coli* K12, within the acceptable ranged from 30 to 70%.

### In silico immune simulation of polyepitope based designed vaccine construct

**Figure 8:**
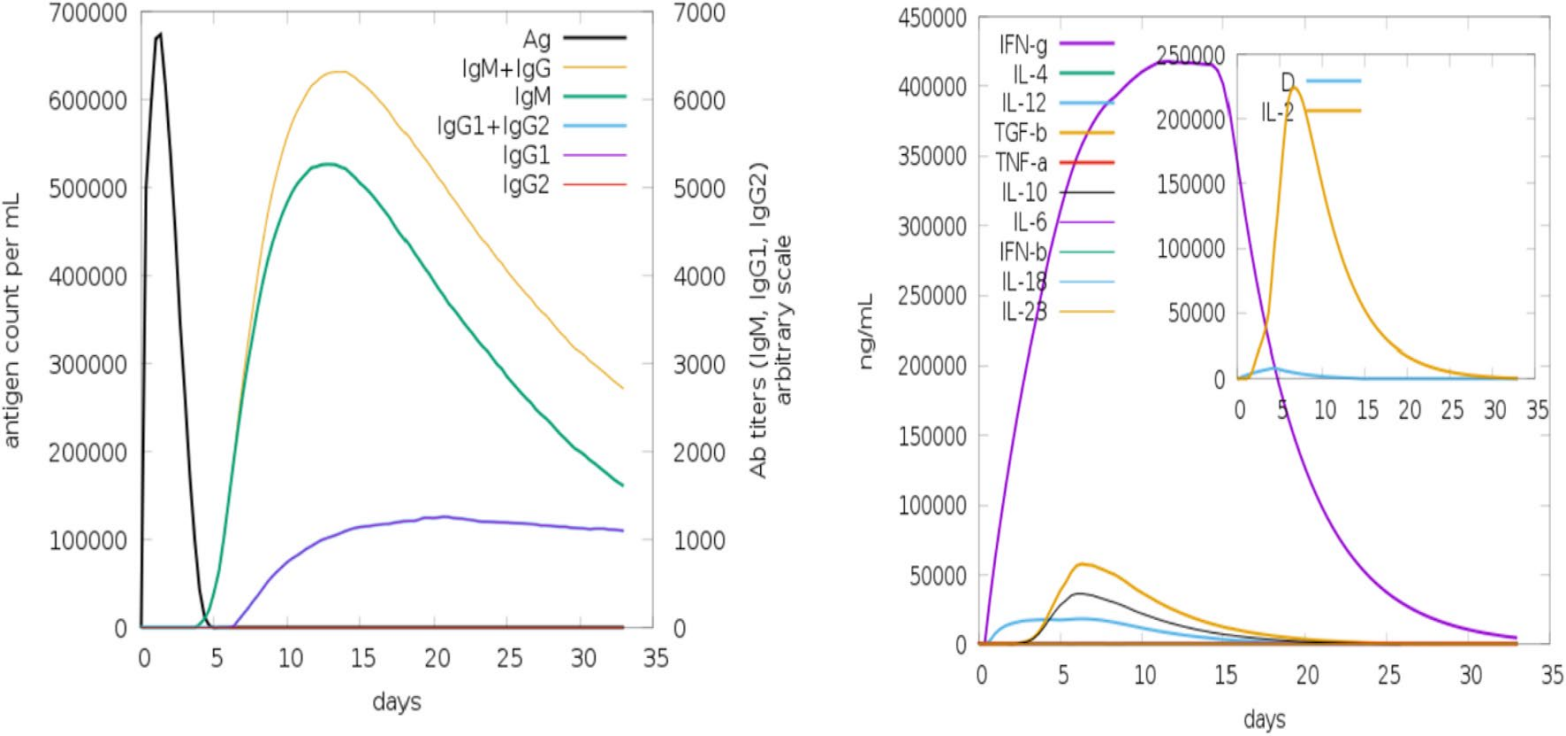
C-immune simulation to verify the host immune system's reaction against the polyepitope-based vaccine engineered. At the left there are antibodies, whereas cytokines and interleukins are shown in right.

**Figure 9:**
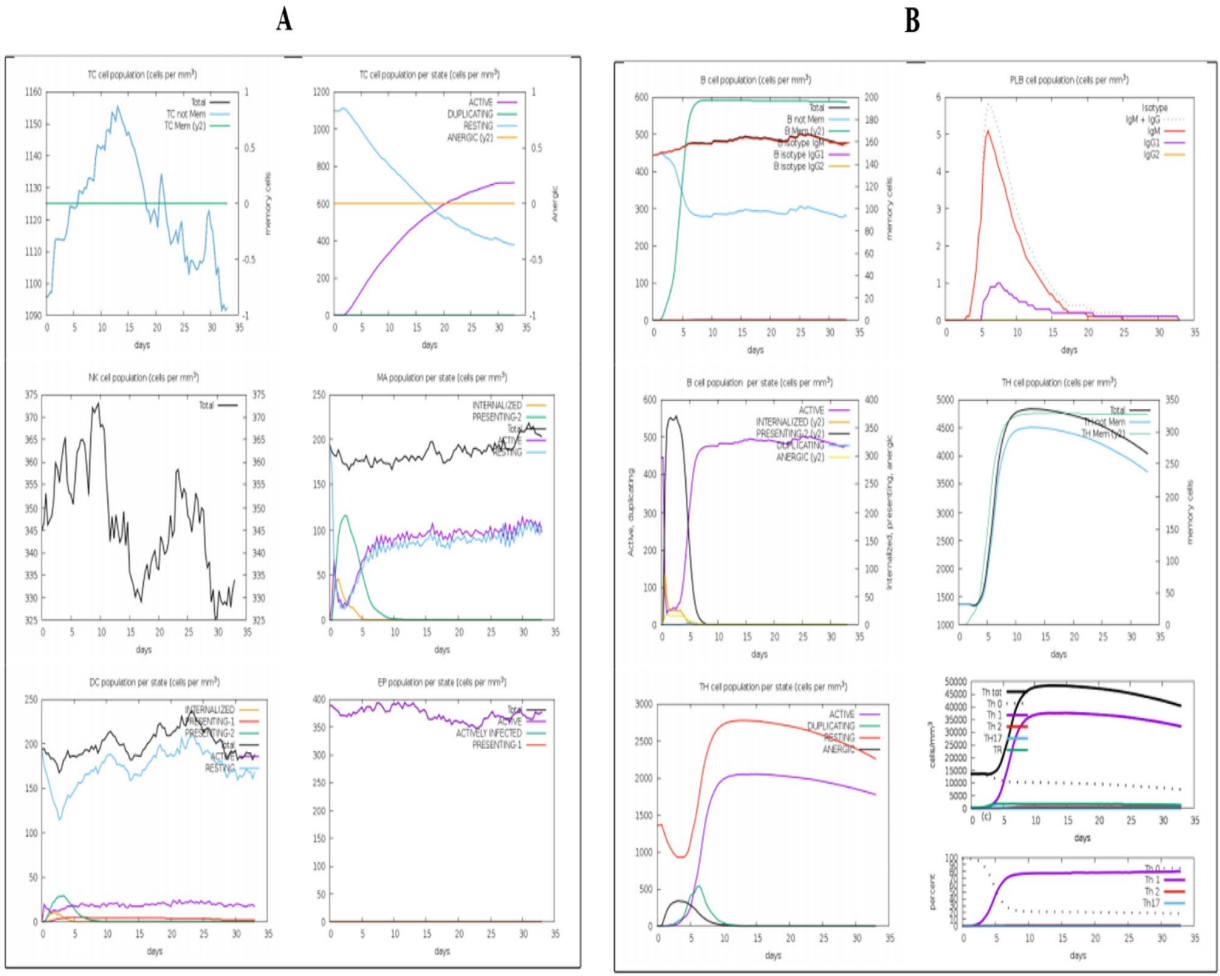
(**A**) Population of B and T cells versus days in response to polyepitope vaccine construct antigen. (**B**) Count of immune cells formed against the response of polyepitope vaccine construct; TC, NK, MA, DC and EP (Cytotoxic T cells, Natural killer cells, Macrophages, Dendritic cells and Epithelial cells).

It appears that both primary and secondary immune responses play a crucial role against the pathogen and considered compatible with the real immune response. Figure 8 depicts the in silico based host immune response towards antigen. The primary response was characterized by high concentrations of IgG + IgG and IgM, accompanied by primary and secondary concentrations of IgM, IgG1 + IgG2 and IgG1 with concomitant antigen reduction. In addition, strong interleukin and cytokine responses have been observed. All of this indicates the host’s successful immune response and removal of pathogens upon successive encounters. In response to the antigen, the elevated B cell population, including memory cells and multiple isotypes, points to long-lasting memory development and isotype flipping. In addition to the cytotoxic T cell, the T helper cell population and their respective memory growth strongly agree with the strong response. In Fig. 9, the different population and per state count of immune cells are given.

### Molecular docking revealed significant vaccine-TLR2 interaction

Molecular docking was carried out between our designed polyepitope-based vaccine and TLR-2 structures. To obtain the active as well as passive residues potentially used for establishing interactions, the CPORT server was used, and yielded information about these residues for each protein (Supplementary file 13). For molecular docking analysis, the advanced HADDOCK guru level system was utilized, with parameters set to default. CPORT-predicted active and passive residues were entered accordingly. Docking led to the clustering of the total 65 structures into 12 groups, which collectively represented 32.5% of the HADDOCK models obtained. The different statistical parameters of each cluster were analyzed and the top cluster having the lowest HADDOCK score (− 16.9 ± 16.9) was identified. Docked structures of this cluster were visualized through chimera and VMD and one representative docked complex was then proceeded towards additional refinements.

**Figure 10:**
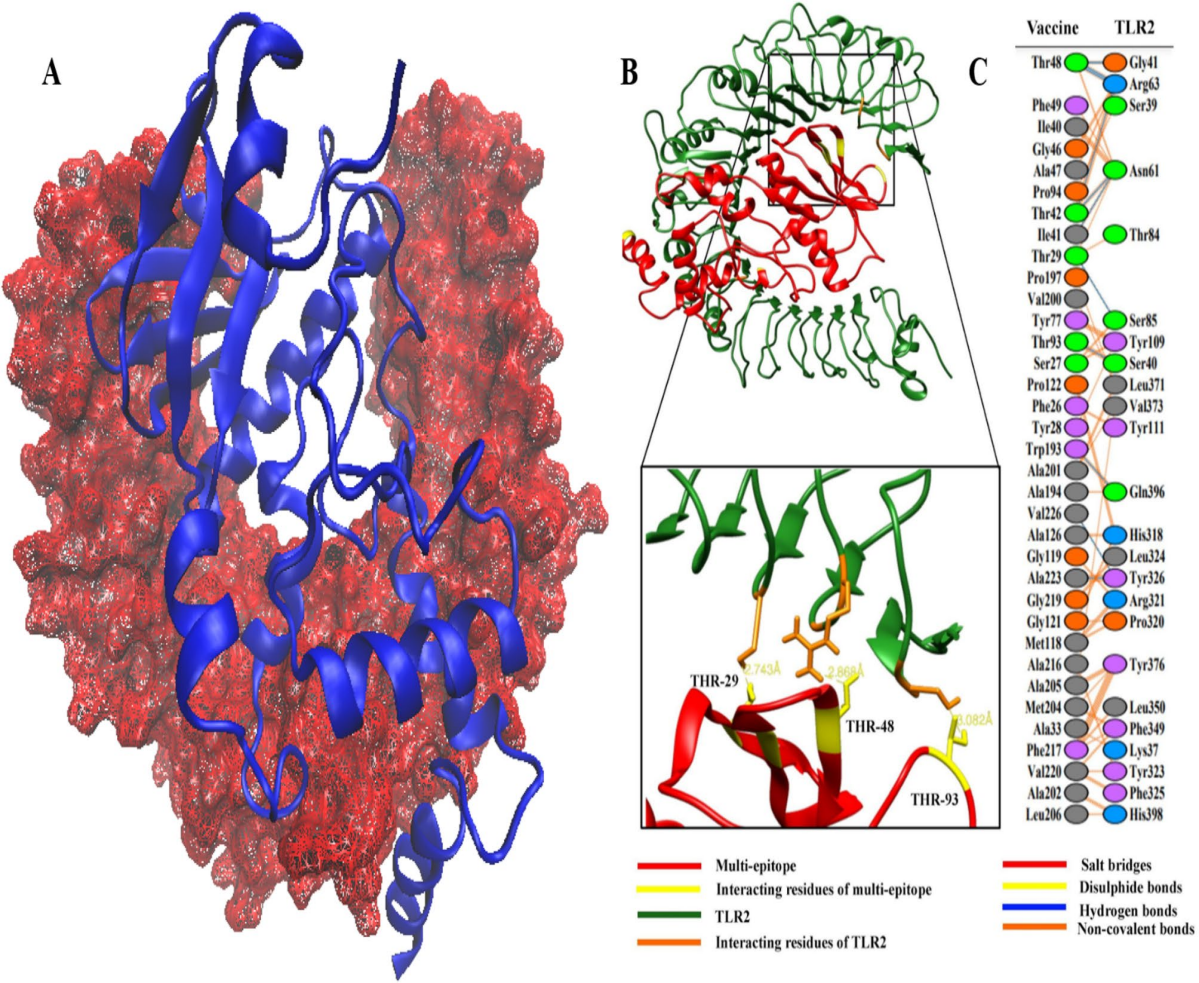
(**A**) Polyepitope vaccine construct (shown in NewCartoon Blue) conformation with respect to TLR2 receptor (shown in wireframe surf Red). (**B**) Analysis of refined vaccine-TLR2 complex at inter-chain level and residue. (**C**) Visualization of some notable inter-chain hydrogen bonds.

Molecular refinements on the docked complex led to the placement of 20 clusters into one cluster which in this case represented 100% of HADDOCK models. This top cluster was observed to have a minimum HADDOCK score of − 164.4 ± 2.6. A docked structure from this cluster was visualized and interactive residues were noted accordingly (Fig. 10). Moreover, PDBsum analysis indicated that a total of 35 residues of the vaccine (with interface area 1616 Å^2^) developed positive interactions with a total of 24 residues of the TLR2 (with interface area 1652 Å^2^) (Fig. 10B). Some notable inter-chain hydrogen bonds were noted and visualized accordingly (Fig. 10C).

### Binding affinity of vaccine-TLR2 complex

Utilizing the PRODIGY server, Gibbs free energy (∆G) was obtained which is a crucial parameter to ascertain whether the vaccine-TLR2 complex formed was thermodynamically possible or not. It was found that ∆G value is − 10.2 kcal mol^−1^ whereas the dissociation constant (K_d_) at 25 °C was calculated to be 3.3E−08. Therefore, this analysis confirmed that the vaccine-TLR complex formed is energetically feasible.

### Molecular dynamics simulation studies

**Figure 11:**
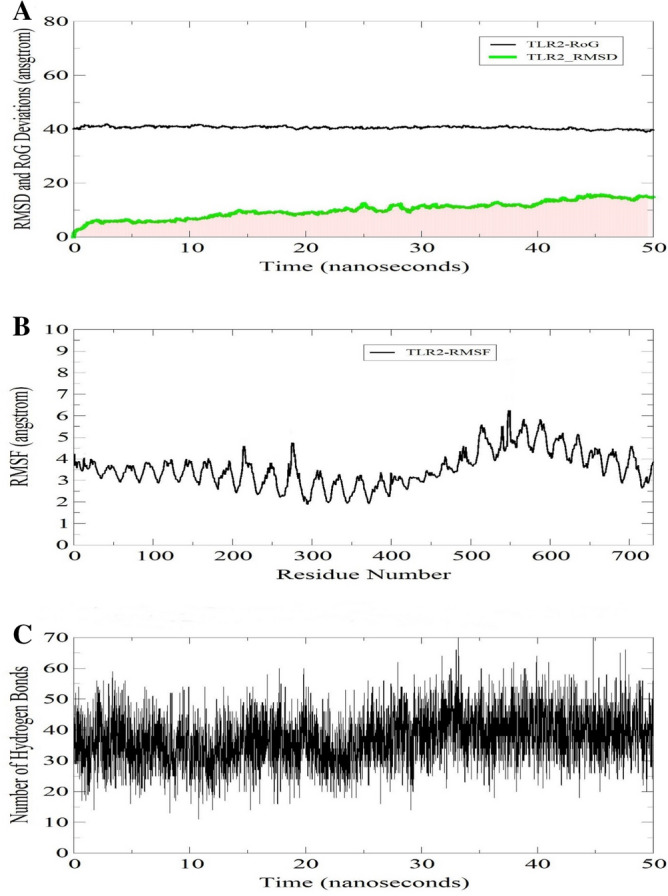
Molecular dynamic simulation of TLR2-vaccine complex. (**A**) Graph showing RMSD and RoG. (**B**) Graph showing fluctuations in RMSF during simulations. (**C**) Hydrogen bond analysis during simulations.

The dynamics and stable conformation of the docked vaccine molecule to TLR2 receptor were probed through a 50 ns run of molecular dynamics simulation. The output trajectories were analyzed through four statistical parameters: Root mean square deviations (RMSD), Radius of gyration (R_g_), Root Mean Square Fluctuations (RMSF) and hydrogen bond analysis. All four parameters are illustrated in Fig. 11. RMSD was calculated for the TLR2 Cα atoms to decipher the structure stability among the superimposed snapshots obtained through simulation. The mean RMSD noticed for the system is 9.9 Å (maximum of 15.9 Å observed at frame 4558 (45.5 ns). Generally, the RMSD plot is showing minor variations, TLR2 structure is very stable and no global conformational changes were seen. Next, *Rg* estimation of the system was done. Rg is a general feature of describing protein compactness and relaxation over simulation time. A higher variation of *Rg* plot implies less compactness and vice versa. The average *Rg* estimated for the system is 40.57 Å (maximum of 42.06 Å) complementing the rmsd findings and affirming stable and compact behavior of the protein in the presence of the vaccine. Furthermore, a general overview of the protein residues fluctuation was concluded from the rmsf calculation. Residues of the TLR2 active residues key in binding vaccine and stabilizing are also elucidated in this assay. The average residue deviation is 3.9 Å (maximum of 6.1 Å). This depicts highly stable behavior of the TLR2 residues after vaccine binding. Furthermore, the strength of intermolecular interactions was determined via hydrogen bonds analysis that demonstrated on average the formation of 37 hydrogen bonds in each frame of the simulation. This highly complies with the strong stability of TLR2-vaccine complex.

### Binding free energy estimation

To affirm the affinity of the vaccine for TLR2, MMGB/PBSA was used as a post-simulation processing to calculate the different free energies of the complex. To achieve this objective, MMPBSA and its counterpart MMGBSA are more considered more precise compared to docking predictions and are less expensive than free energy perturbation. Both methods are efficient program as an end state free energy calculation method. The different binding free-energies calculated in MMGBSA and MMPBSA are presented in Tables 2 and 3 respectively. The analysis revealed a net delta energy of − 43.6643 kcal/mol in MMGBSA and − 73.4728 kcal/mol in MMPBSA. In MMGBSA, delta energy revealed for the complex, TLR2, and vaccine is − 25,678.4794 kcal/mol, − 22,076.1979 kcal/mol, and − 3558.6172 kcal/mol, respectively. From the total MMPBSA energy, the vaccine binding energy is − 3451.8828 kcal/mol. The complex binding energy is − 25,356.1860 kcal/mol, TLR2 binding energy is − 21,830.8304 kcal/mol. In both methods, the net electrostatic energy contributes favourably to the net binding energy. The net electrostatic energy of − 312.9003 kcal/mol is estimated in both MMGBSA and MMPBSA for the system. The van der Waals energy is − 93.5882 kcal/mol (complex = − 10,522.8197 kcal/mol, TLR2 = − 8786.4960 kcal/mol, and vaccine = − 1642.7355 kcal/mol). The net solvation free energy is revealed non-favourable to the total energy in MMGBSA (301.5696 kcal/mol) and MMPBSA (271.7610 kcal/mol).

**Table 2:** MMGBSA based binding free energies of the complex, TLR2, vaccine and net delta energy in kcal/mol.

Energy component	Average	SD	SE of mean
**Generalized born**			
*Complex*			
Van der Waals	− 10,522.8197	53.7628	5.3763
Electrostatic	− 95,157.9075	174.7182	17.4718
Polar solvation energy	− 16,023.2839	140.2591	14.0259
Non-polar solvation energy	472.8864	4.4365	0.4437
Gas phase energy	− 10,128.0819	196.1939	19.6194
Solvation energy	− 15,550.3975	138.8135	13.8813
Total	− 25,678.4794	148.6605	14.8661
*TLR2 receptor*			
Van der Waals	− 8786.4960	51.1912	5.1191
Electrostatic	− 77,040.1613	169.8608	16.9861
Polar solvation energy	− 12,863.0115	145.4925	14.5492
Non-polar solvation energy	358.4210	3.9722	0.3972
Gas phase energy	− 9571.6074	178.9572	17.8957
Solvation energy	− 12,504.5906	144.2641	14.4264
Total	− 22,076.1979	124.3022	12.4302
*Vaccine*			
Van der Waals	− 1642.7355	23.7365	2.3737
Electrostatic	− 17,804.8459	97.0236	9.7024
Polar solvation energy	− 3475.5293	81.4546	8.1455
Non-polar solvation energy	128.1528	2.6121	0.2612
Gas phase energy	− 211.2407	94.4941	9.4494
Solvation energy	− 3347.3765	80.1900	8.0190
Total	− 3558.6172	51.8689	5.1869
*Differences (Complex—TLR2–Vaccine)*			
Van der Waals	− 93.5882	9.9121	0.9912
Electrostatic	− 312.9003	46.2789	4.6279
Polar solvation energy	315.2570	43.5847	4.3585
Non-polar solvation energy	− 13.6874	1.3925	0.1393
Gas phase energy	− 345.2339	45.0664	4.5066
Solvation energy	301.5696	43.2835	4.3284
Total	− 43.6643	6.8491	0.6849

**Table 3:** MMPBSA based binding free energies of the complex, TLR2, vaccine and net delta energy in kcal/mol.

Energy component	Average	SD	SE of mean
**Poisson Boltzmann**			
*Complex*			
Van der Waals	− 10,522.8197	53.7628	5.3763
Electrostatic	− 95,157.9075	174.7182	17.4718
Polar solvation energy	− 15,551.4301	134.6322	13.4632
Non-polar solvation energy	323.3260	1.7048	0.1705
Gas phase energy	− 10,128.0819	196.1939	19.6194
Solvation energy	− 15,228.1041	134.0287	13.4029
Total	− 25,356.1860	145.5654	14.5565
*TLR2 receptor*			
Van der Waals	− 8786.4960	51.1912	5.1191
Electrostatic	− 77,040.1613	169.8608	16.9861
Polar solvation energy	− 12,507.7310	140.3346	14.0335
Non-polar solvation energy	248.5080	1.1626	0.1163
Gas phase energy	− 9571.6074	178.9572	17.8957
Solvation energy	− 12,259.2230	139.9714	13.9971
Total	− 21,830.8304	123.7853	12.3785
*Vaccine molecule*			
Van der Waals	− 1642.7355	23.7365	2.3737
Electrostatic	− 17,804.8459	97.0236	9.7024
Polar solvation energy	− 3326.9413	82.7583	8.2758
Non-polar solvation energy	86.2992	1.6065	0.1607
Gas phase energy	− 211.2407	94.4941	9.4494
Solvation energy	− 3240.6421	81.9779	8.1978
Total	− 3451.8828	54.2440	5.4244
*Differences (Complex—TLR2–Vaccine)*			
Van der Waals	− 93.5882	9.9121	0.9912
Electrostatic	− 312.9003	46.2789	4.6279
Polar solvation energy	283.2422	44.5670	4.4567
Non-polar solvation Energy	− 11.4811	1.2082	0.1208
Gas phase energy	− 345.2339	45.0664	4.5066
Solvation energy	271.7610	44.3420	4.4342
Total	− 73.4728	9.1691	0.9169

## Discussion

While *M. abscessus* infection may not represent an immediate threat, medical attention on this opportunistic pathogen is only growing with time, and rightfully so. There is still time and a window of opportunity to manage these bacterial infections by developing effective therapies such as vaccines before the emergence of more drug resistant strains of *M. abscessus* which is inevitably occurring due to the widespread and uncontrolled use of antibiotics.

The ratio of the core and pan-genome sizes was found to be 0.34. It means that the core genome is approximately 34% of the pangenome. This indicates *M. abscessus* has an open pangenome which will continue to receive new genes. It is possible that horizontal gene transfer has a crucial role in the evolution of this emerging microorganism thus enabling it to acquire increasing genetic material. In our study, it was found that bacterial isolates from different areas of the world clustered together i.e., from the USA to Malaysia, Russia, and China. This observation indicates that the global dissemination of *M. abscessus* is ongoing. Exploring the phylogeny results confirmed that pangenome phylogeny (Fig. 2) provided better resolution to discriminate between the bacterial strains vis-a-vis the core phylogeny. The pangenome based tree clearly separated closely related strains that were otherwise depicted phylogenetically closer in the core genome phylogeny. Apart from that, useful insights were drawn from the genomics data. The variation in genome size, GC content and protein coding genes within *M. abscessus* strains make clear physiological and genomic differences between them and the elaborated genetic makeup of the species in the natural environment/niche.

Apart from genome analysis of *M. abscessus*, we took an aim to design a multi-epitope vaccine against this opportunistic pathogen. A reverse vaccinology pipeline Vaxign was used to select exoproteome and secretome-related proteins as targets localized in these regions have the increased tendency to act as antigens^42^. However, to overcome the limitation of this tool, more filters (antigenicity, physicochemical characterization, transmembrane helices less than two) were also considered to prioritize antigens for vaccine design.

Epitope mapping and selection was conducted to identify immunogenic regions within the four core antigenic proteins. It was found that the selected T-cell epitopes were conserved and antigenic so they might be useful components of a broad-spectrum vaccine. The prediction of discontinuous B-cell epitopes was carried out on the three-dimensional structure of multi-epitope vaccine to validate the presence of B-cell epitopes and remove false positive hits. There is a growing consensus that B-cell epitopes are mostly conformational in nature as opposed to being linear^43–45^. Thus, it was reasoned that sequence-based analysis is not suitable to identify potential B-cell epitopes. That is why an endeavor was made to first obtain the high quality and stabilized structural model of the polyepitope vaccine and then use it as input for programs like Ellipro and DISCOTOPE for 3D structure-based epitope prediction.

Encouraging results were obtained in case of conformational B-cell epitope mapping within the designed multi-epitope vaccine. This development indicated that besides activating T-cell response, our designed vaccine will serve to activate B-cells too and lead to the induction of specific humoral responses. Indeed, vaccines that activate both arms of adaptive immunity are likely to resolve microbial infections due to the emphasis on both the cellular and humoral aspects of immunity^46^. However, the major focus of this study was on the activation of T-cell immunity. Vaccine-elicited T-cell responses, in addition to being durable, can circumvent the antigenic drift that in turn is linked to the evasion of antibody memory responses^47^.

The multi-epitope vaccine showed good physicochemical features in terms of thermodynamic feasibility, stability, hydrophilicity and expression capacity. The multi-epitope vaccine is non-allergen, thus harmful responses of the vaccine are not expected. The vaccine designed in the present study exhibited a high level of antigenicity which is much preferred for immunological applications. In addition, overexpressed of this vaccine could be done in *Escherichia coli* K12 strain.

Altogether, the predictive framework for multi-epitope design and its downstream analysis using molecular docking and interaction analysis is a good starting point for the development of vaccine candidates. However, further in vitro and in vivo studies are necessary to establish whether the epitopes and multi-epitope vaccine proposed in this study will confer protective immunity against *M. abscessus* infection or not.

## Conclusions

*M. abscessus* is an emerging multi-drug resistant infectious pathogen and has the potential to exert serious infections in humans. Development of a vaccine that could protect humans from infections of the said pathogen would be a breakthrough. Due to the complex biology of the pathogen and the daunting nature of conventional vaccine development, vaccine development against this pathogen is challenging. Therefore, we herein formulate an in silico vaccine based on antigenic epitopes collected from across the pathogen’s core antigenic proteins to develop an effective vaccine candidate. The selected epitopes were selected from the core genome thus enhancing the broad-spectrum of the designed vaccine ensemble. The vaccine has also demonstrated to elicit both major humoral and cellular immunity. Notably, the included epitopes in the vaccine construct are non-homologous to the human host, antigenic, and non-allergic. The vaccine ensemble itself possesses all good qualities needed for a practical vaccine candidate and is showing high affinity and stability of binding to TLR2 innate immune receptor, the interaction with which is vital in recognition and processing by the host immune system. The use of all these immunoinformatics and biophysical approaches are indeed extremely useful in guiding experimental studies and saving time and cost. However, because of the limitation of these used tools and servers, in vitro immunological assays are recommended to evaluate the biological protection efficacy of the vaccine.

## Supplementary Information

Supplementary Information 1.

Supplementary Information 2.

Supplementary Information 3.

Supplementary Information 4.

Supplementary Information 5.

Supplementary Information 6.

Supplementary Information 7.

Supplementary Information 8.

Supplementary Information 9.

Supplementary Information 10.

Supplementary Information 11.

Supplementary Information 12.

Supplementary Information 13.

Supplementary Information 14.
