# Supplementary material for: Designing a multi-epitope vaccine against Mycobacteroides abscessus by pangenome-reverse vaccinology
Source: Sci Rep. 2021 May 27;11:11197. doi: 10.1038/s41598-021-90868-2 (PMC8159972; doi:10.1038/s41598-021-90868-2)
Supplement: Supplementary file 2 — Supplementary Information 2. [file 41598_2021_90868_MOESM2_ESM.docx]

**Designing a multi-epitope vaccine against *Mycobacteroides abscessus* by Pangenome-reverse vaccinology**

Hamza Arshad Dar^1#^, Saba Ismail^1#^, Yasir Waheed^1*^, Sajjad Ahmad^1^, Zubia Jamil^1^, Hafsa Aziz^2^, Helal F. Hetta^3,4^, Khalid Muhammad^5*^.

1. Foundation University Medical College, Foundation University Islamabad, DHA-I, Islamabad 44000, Pakistan
2. Nuclear Medicine, Oncology, and Radiotherapy Institute, Islamabad 44000, Pakistan.
3. Department of Internal Medicine, University of Cincinnati College of Medicine, 231 Albert Sabin Way, Cincinnati, OH 45267-0595, USA.
4. Department of Medical Microbiology and Immunology, Faculty of Medicine, Assiut University, Assiut 71515, Egypt.
5. Department of Biology, College of Science, United Arab Emirates University, Al Ain, 15551, United Arab Emirates.

*Correspondence: [yasir.waheed@fui.edu.pk](mailto:yasir.waheed@fui.edu.pk)*,* [*k.muhammad@uaeu.ac.ae*](mailto:k.muhammad@uaeu.ac.ae)

>CORE_REP|Org2_Gene442#

MRALTRALTIASIALLASAGCAGDMTSRPGGAAEPCGIAANGVPITKTAATQAATERNISTNPEVATGFRTGMTAVRTASYAAATANPLATQAACKVLRDGGSAADAVVAAQAVLGLVEPQSSGIGGGGFLVYYDAASGAVQTYDGREVAPAAATENYLRWVDNADRTAPRPDVRSSGRSIGVPGILRMLHDVHTEHGATPWRDLFTPAVQLADDGFEISPRLAAAITDSAASLALDPEADLYLLNTDGSAKPAGAKITNPAYAKTLGAVATSGPDAFYRGAIAEAIVAAAADTSAGRTPSLMTTADLSGYTAKRRAALCTTYRDHEICGMPAPSSGGIAVAATLGILQNFPMASFRPTDIDLDGGKPTVDGVHYIAEAERLAYADRDKYVADTDFVALPGNSPDTLLNPTYLAGRARLISGDTTMGIAKPGEFNIPASAAVSTPEHGTSQVTVVDSRGNAASLTTTVESAFGSFHMVDGFFLNNQLTDFSAEPVGKDGLPVANRLQPGKRPRSSMAPTLVFERAESGKRGALLLALGSPGGSVIIQFVVKTLVGILDWGLDPQQAVSMVDFGANNSPKTNVGGEHPNIDARDNGADDPLIQRLRARGHTVDVADQSSGLSALVRRGPGWIGGADPRREGLVMGDTG

>CORE_REP|Org17_Gene1124#

MSSPAAVPVKPEADTDFDVLIVGSGFGGSVTAMRLTEKGYRVGVLEAGRRFADEEFAETSWDLRKFLWAPMFKCFGIQRIHLLSNCMILAGAGVGGGSLNYANTLYVPPDPFFNDPQWKGITDWRAELSPHYEQAQRMLGVVKNPTFTDADRLIKEVADDMGAGDTFVATPVGVYFGPDGTKTPGVKVPDPYFGGAGPDRVGCTECGSCMTGCRVGAKNTLVKNYLGLAESNGAQVIPLTTVTAVQQGADGVWRVSTKSTGRWVRKQRKTYTAKYVVLAAGTWGTQNLLFKMKDKGLLPKLSQRLGVLTRTNSESIVGAGRLEYKDDLDLTHGVAITSSFHPTSDTHIEPVRYGKGSNAMGLLQTLMTDGDGPKSRWRQLIENARADWRGTFRMFNVTQWSERTVIALVMQHLDNSITTFTKKTLFWRRLDSKQGHGEPNPTWIPAGNEATRRLAAKIDGVAGGTWGELFNIPLTAHFLGGAVIGTSPENGVIDPYHRVYGYPTMYVVDGASISANLGVNPSLSITAQAERAASLWPNKGETDLRPEQGLPYQRMAPVPPVHPVVPADAPGALRRLPIEPVSSAS

>CORE_REP|Org8_Gene2190#

MDYDYDVVVIGSGFGGSVAALRLTEKGYRVGVLDMGKRWKREDFPPNNWHVRKAMWAPALGCYGPQRLTVLGKTFIASAVGVGGGSLIYGNTLYEPLEQFYVDKQWAHISDWKSELAPYYDQASRMLGVAQTPHTTPPDEVLLAVAKDLGVEDTYHPTNVGVFFGDEPGKTVPDPFFGGAGPERTGCIGCAACMTGCKHNAKNTTETNYLYLAEHAGAQIHPLTQVLDVRPLEDGEGYAVSTKQTGRLLRKKKRTFTAQHVVFSAASLGTQRLLHKFKDSGSLPNLSERLGEQTRTNSESVPIVYSPTRDDFSQGVAITSSIHPEPNTHVEVVRYGEGSTFLSMLGTNMVDGGPWRFARTWLANLRHPAMMLRSMFPYRAAQHSIIVLVMQSLDNSLTTYLKRGMFGKKMTAKQGSGEPNPDWIPVAHDVARRMADKVDGYAGSTHLDSMNIPLTAHFIGGCPIGDSPETGVIDPYQRIYGHPGLHICDGSAISANLGVNPSFTITAQAERAMAFWPNKGEADPRPALGSAYRRVAPVQPNHPTVPESAPGALRLPLTPV

>CORE_REP|Org29_Gene3335#

MANKWDIEALRGEGLQAIANSQNYVTAAIRGNGKSPVTITNPDLTANERQLFDWYDMDAGMDLNTLGGDLELFKNATATMKAAAERQHGQLQRLIGLWEGKGSESANDFLKTHNSTADAVTDEFGKVSTGLDGLRNALWNIVDLKKQASTMVDGLVTDRTHFDSAVATYKTGMGDKSQADETNATMIGPHVKNNIEGQLLPAFKKAWSAGGGAYDTLINGLKQELPPDFKLPPGVFGPDYDTTDEPAKTTKGKGKQDDKDGGETSGESGESGNSGVNSGAGGGTASGGMQGTATPASATGNAGGQLSGAGQQQGAGQGQQGMDPSQMLSGMTGALTGALSSIGQAASGIVSAITEGISSIPFDQMGQGLGDDQFDGRADEAADKKDEAAADGKKDPDAKMAAAKDAAIEEARADSGATFATDGKPAPGIQLAGAGGLEATPTAAPGQTTPGAPLGATPTGTIPPAAGGLSAAQPAGSSASLTPHPVQAQPPTVPQHPEPQSAAARQPSPLPSVGPTDASAQPQEAKTEAGETPCEIAADELPKAGR

>CORE_REP|Org18_Gene2441#

MATNRVLYAVAVSAACALATVTACDRDAAEAPPRSAPQAGSEEAVGFAHSLHEKVTVDNVVKHLSALQEIADKNNNTRAAGTAGFDQSVDYVVKALKDKGFDVQTPEFSFKYFQAKSLDLTVGPKKVDAGVLSYSPGGRVEGRLVPARAEESPGCTVEDYDGLEVKGAVVLVDRGSCPFADKERVAAERGAAAVIIADNVDENKTSGTLGEDSSPKIPVVSVTKSVGADLRAHPDKVVLNVDAETKDVKARNVIAQTKTGATTDVVMAGAHLDSVPEGPGINDNGTGTAAVLETALQLGPSPDVKNAVRFAFWGAEEEGLIGSTDYVKSLDVAALKNIALYLNYDMLGSPNAAYLTYDGDQSDEPDPNEVPVRIPEGSAGIERTEVAYLAEQGKKAHDTGYDGRSDYDAFSRAGIPTGGIFSGAEDKMSDEEAKQWGGKAGQPFDPNYHQAGDTLANVNKDALKINAGGVAYTVGLYAQSIDGRNGVPVHEDRTRHQLKG

>CORE_REP|Org31_Gene3714#

MKRTVNDRFAVRGKRTVATALMVPPLMVAGLMMFPTTVAVTSAEPNDMASLITQLADTNQQIEQLTADVQTQQESINKGLVDLQAARDNAASAAAQVAEGQRAVDAANGAIEEAQGKFDRMAAATYMAGPSTSYLTATNPDDVVRLASVTKSVEASSQTVMDNLRRARTEQVNKQSQARAIQEKADQAAADAQQQQDDLVSAMKDVQKKLEAQRGVAADLTAKKKSAEAQLAAARGPAYAASTATARVINPSAAIAGNGNEWTEGPAPVSSGGQWDTTLPMIASANVPTDPTQTINMVLGIGNTAANVGQSAVCGVIGIFCPKAAPAAAASGEGGEYLPKVYGRENVERVIARAGSALGTPYSWGGGSYNGPTRGIDSGAGTVGYDCSGLMMYGFAAVGIRLRHYTGYQYNSGRKVPSAQMKRGDMIFYGPNASQHVALYLGNGQMLEAPNTGDVVKVSPVRTSGMTPYVTRMIEW

>CORE_REP|Org25_Gene4667#

MVRLRKLAVAASAILVAGCGMGRPPGAIDGEYLTVGTTDRVSTLDPAGAYDNGSFQVENQVYPFLMNFTPGTGDLKPDLAAGCGFENPTLYRCTLKPGSVFANGHELTSSDVKFSYDRERVINDPNGPQSLLANLDRVETPDDLTVDFRLKLPNDQTFPQVLATNAGPVVDEEVFPPDRLLDDDAIARAEPFAGPYTITSHTKNQLIGLRANPKYVGDLGKPQWDLIGIKYYTGGENLKIDIENRAIDVAYRSLSPNDIETLRVNPRLSVHEGPGGELRYIVFNLKTMPGATDAQKLAIRKAVASLVDREALSRDVYKGVYTPVYSVVPESMTGSVESFKRLYGVKPNVDLARKFLSDAQIATPVLINLQYNPDHYGGNSSEEYAAVKGQLEASGLFRVDLQSTEWVAYQERRSSDSYPVYQFGWFPDFPDPDNYLTPFFMPDNMLVNHFQNDTITRLITAEVTEPDSAKRLRIIGQIQELMARDYISTLPLLTGKQIAVSVKNVDGIKLGPSFKFQFTPLKKTGGTA

>CORE_REP|Org32_Gene4700#

MSKAQVEQTDVALIGSGIMSATLGALLRLVEPDLSITLIERLDAAASESSDPWNNAGTGHSALCELNYTPQKADGSIDITKAITVNEQFQVSRQFWAYAVENGVLPDVRGFLNPIPHVSYVHGADKVEFLRKRHEALVGNPLFAKMEFINDDDEFARRLPLMAAGRDFADPVGLNWSQDGTDVDFGELSKQLVGFGVRSGTSAVFGTEVRNISRESDGGWLLKLVNGRTGEKRKLKAKFVFVGAGGGALGLLQKAGIPEAKGFGGFPVSGAFLRTNSTDLTDGHKAKVYGFPPLGAPPMSAPHLDTRVINGKEWLLFGPFAGWSPKFLKMGKVTDLPASIKPNNLLSMVGVGLTEFKLATFLLSQLALTPDDRIDMLREFAPTAQRADWELITAGQRVQVIRPAKGKGGALEFGTMVLNSADGSIAGLLGASPGASTAVPAMLDVMQRCFPDRYSNWMPKLKEMIPSLGISLSDEPSLFGEVWDWGTRVLGLEQ

>CORE_REP|Org30_Gene3943#

MVTARALDPRHWRRSTHALLAVAVVLAIAALVLLASLTTGASTAHQLQSADPEPALITPAPGVVPVSDSAPIPTADGLAQALDRALSDPALGMFTGRVTDALTGRELWQQGSTVPMVPASTNKVLTAAAALLTLERDAKLTTSVVADTAGQRGLVTLVGGGDPIVSAAPAGTDTWYRDAARISDLADQVRKSGVAVTSITVDISRFGGPSMAPGWDPADIEGGDVAPIQAVMLDAGRTQPTDYDSRRSTTPALDAGRALAAALGVDPEAVRLDAAPPTAKSIASVQSAPLMERLREMMNASDNVMAETIGREVALATGRAQTFSGTVDAVTSQLRSAGIDLTDLTLRDSSGLSVDDRVTARTLDEVIGAAAGPDKPKLRPLLDVLPIAGGSGTLSERFVTQNQTSAGWLRAKTGSLTGVNTLAGVVTDVSGRVLTFSLMQNHATAPTARNAVDNTAAVLRSCGCS

>CORE_REP|Org17_Gene692#

MSTGLNRRKLLGAAGVTAAVAGAAGAGVLGGRASAASTGPVNVKVPFRGDHQAGIVTPAQDRMHFCAFDVMPNATRGEVQAMLRQWTEMADRMTRGEETTSGGAVDGNPYSPPTDTGEALDLAASALTLTIGFGPSFFRKDGVDRFGIADKLPPPLQELPKFRNEKLDPARCGGDICIQACADDPQVAVHAIRNLARVGFGTVAVKWSQLGFGRTSSTSRSQVTPRNLFGFKDGTRNIKAEDTAKVDSSVWVAKGDDPAWMAGGTYLVARRIRMLIESWDRTVLTEQERVIGRAKGSGAPIGQADEFAALDFTGKKPDGEPLLDVDSHVRLASAEELGGIEILRRGYNFTDGSDGFGHLDAGLFFIAFVRNPQTQFIPMQRKLATEDALNEYILHTGSAIFACPPGLGPKEYWGQALFG

>CORE_REP|Org10_Gene4433#

MTKESRNMDVNRRNFLRGAAVGAAGTAITGAALVKGAEVDANAAAVAVPPSRYPFHGAHQSGILVPPPAEKQNFACHVAFDVTSKNKDAVVAAFKTLTARARFLCDGGTPPDLGIGEPPADSAVLGPVVESDGLTVTVAVGSSLFDRRFGLADRKPAKLKPMTVFPNDFPEAAWSHGDLLVQLCAHNPDTVHHALRDITRAVRGDLQMRWRIEGYNSPPRPSGTGRNLLGFKDGTANPVSDDAEKLVWTGDGEPAWTVGGTYMVVRLIRMMVEFWDRVSINEQERMFGRRRDSGAPLDGNNEFDTPNYAADADGKTIPLDAHIRLANPRTADTDNQRLVRRSYNYDLGVDANGNMQSGHVFVCYQQDLERQFETVQNRLNDEPLIDYVQPFGGGYFFALPGIADEKDWYGRALLG

>CORE_REP|Org7_Gene3229#

MPGLDSTFDAALAPIRAAVDDRILAGAVTLVWQGGHLRHLGATGYRDIDAGLSMAENTIFRIASMTKPIISAATMSLVDDGAIRLSDPITTWLPEFSDMRVLKDPEGPLDDTFRAPRLITVEDLLTHRSGLTYDFISTGPIAKAYHPLHTAAFSEPDEWLAAIAALPLVYPPGERFHYSHSTDVLGLLIARAAGVPLNTLLRQRILDPLGMNDTDFFVPEHKTARLARLYGLGDDDTIVAADSGYLTAMPTSAPALCRGGGALASTAHDYLTFARALLGGGQADGVRILSPESTQALRTNRLTPAQRRLPSFGIPYWTGRGFGLGLSVVMDPNEAALFGPGGTGTFGWPGAFGTWWHADPKADAILMFLPQWRMPELDPKAALARTSTIRLQLLHVQFGQAVYAAL

>CORE_REP|Org1_Gene2259#

MSSRSLSRRGLIVGGGAAAALTAGAGLSAWSAQSHAARPSAERQVEPFYGDHQAGIATAPQAHAMFLALDLLSAADADMGTARENLRSILRLWTTDAARLTQGIPALADTEPELATTTARLTVSAGLGPSVFAKTGLADRCPASARDFPAFSTDRLDKRWCGGDLLLQICADSPLLVAHAARVLLKNVRSLASERWRQTGFRTPRPEDPSGGTMRNLMGQVDGTVNPTAPELDSLLWHQGEDHEWLKGGTLLVLRRITMNLDGWDQLDRKLRDLVMGRRSDNGAPLSGEKESDEPDLTMTRGGIPVIPATSHVALARHRNPHERFLRRPYNFDDAPLPGTSSNAGLIFAAYQRDIASQFVPVQQRLAEKDEFNQWNTAVGSAVFVMPPGTTEDGYLGRSLLG

>CORE_REP|Org7_Gene3034#

MPELSTRCFDESSLMRRILSIVLTIALLVSACGKHSAIVVAELDPQTTAKLDSAINEILTSAAVPGAIVGVWGPKGQYVRTFGVANTATRAPLRPDFYHRIGSVTKTFTVTAVLQLVDEGKVELDDPIAKYVDKVPNGNKITLRELARMQSGLFNYSMSLAFSRDLEADPRRQYSTHELLDYAFAQPSNFVPGQGYEYSNTNAVLLGQVVEKVSGQTLPAFVQEHITGPLGMRHTSFPDTNAIPDPHARGYTQLTPTGSLVDSTDWNPSWASWAGAMISTLDDLRIWVPALATGVLLKPDTQQQRLQTVAMPPVPDDIRYGLGIFDARGWIGHNGSIPGYQTLAIYSPAEQTTVVALLNTDVAKQPAQPSTLFGTAITKVISPDHVFLLENAIPQPR

>CORE_REP|Org29_Gene536#

MTTGASNIRHGDRGPAVTEVREVLTALGFLEDPDEVLATGRHVMVDRFDATLDDAVRAFQQCRGLLVDGIVGPATYRTLKEASYRLGARTLFHQFSAPMYGDDVATLQKRLQDLGFYTGLVDGNFGLQTYNSLMSYQREYGLTADGICGPETLRSFQLLGRHVTGGSAHAIRETEHVRNAGPQLSGKRIVIDPGLGGGDRGRIVPGREGPTSEADILWDLASRLEGRMTAIGMDTYISRAIQNNPTDVERATYANNVGADLMISLRFDAQPTVAASGVASYHFGNLHGSVSTIGHMLADFIQREVAARTGLRDCRAHGRTWDLLRLTRMPTVQVDIGYITSPHDVSILSSAHYRDVVAESILAAVKRVYLLGKNDRPTGTFTFDELLAHELSAGS

>CORE_REP|Org10_Gene1299#

MNALTKLNATRSPLLRALVAALLVVIGAGGGYAIAAHKTLTLNVDGNAMTVTTVKSRVSEVLADYGYALSDRDDVAPAKHDSVRDGDTIVLKRSRPLDISVDGQDTQQVWTTASTVNDALSQLSMTDTAPTAATRGSRLPLEGMSLAVVSAKTVQLNDGGVISTPRIAAPTVGALLEATGNPLQQFDTVDPAPSTPVTADMPITVTRIRVSKVTEQAPLAPTPQKIEDPEMNMSRSVVQDPGAPGTQDIVYSVLSVNGRETGRIPVSNNVLTPARDSVLRVGAKPGTEVPAVTRGSAWDALAQCEAGGNWAINTGNGFYGGVQFDYGTWLAHGGGKYAPRADLATREEQIAIAEKTLSAQGWGAWPVCSARVGAR

>CORE_REP|Org11_Gene1319#

MRVGIPTEIKNNEYRVATTPAGVAELTRRGHEVIIQSGAGDGSSISDVDFKAAGAQVINGAEQVWAEADLLLKVKEPIEPEYALMRRGQTLFTYLHLAASLPCTQALLKSETTSIAYETVQTSDGALPLLAPMSEVAGRLAAQVGAYHLMTPVGGRGLVMGGVPGVGPADVVVIGGGVAGYNAARIAAGMGAHVTVFDVNLGKLRELDGEFGGTIRTRYSSTMELEGAVKRADLVIGAVLIPGAKAPKLISNSLVSQMKPGSVLVDIAIDQGGCFADSRPTTHDEPTYTVHDSVFYCVANMPGAVPRTSTFALTNATMPYVLALADKGWRQACRDDAALAQGLSTHEGALLNTHVAHDLGLAFTDPAEVLG

>CORE_REP|Org28_Gene2648#

MTQHRAIRTRGGRGAELSENEITNIVPIDEFGLSDALERPELEDYDVYEDAPEEGAETEDQTALDQLEQDWEEDTDEADRIRASIEATADWFAASAARTRDPRDTPTDKLPRISSGGVHRRREIGQVGKTRLALAAMAAGAAAAAGYNALSEHDAATAADHHGLALGNSAAVLATHGPQLVSAPLATDASVTDEQLAKATAFATERADREKRLLAPRFVMPTNGTFTSGFGYRWGALHGGIDIANSIGTPIVAAADGVVIATGPTAGYGAWVKIRHSDGTVTLYGHINTWEVSVGQRVMAGDRIATIGNRGNSTGPHLHFEVLLGGSQRIDPQGWLANKGLTFTRFGD

>CORE_REP|Org24_Gene1824#

MCQRFIIPPAVLSRLAEDGDIAEDSRAALSATAACELSWRTLRNAHTRATQASLLTSRAAALGAATTGLAKIPETPVFDCRQTTSLPGVAVADPATSKDATAQRAFDETAAVAQFYRNCFGRNSVDNAGMTLVSSIHYGVKYSNAFWNGSQMAYGDGDGQIFLDFTKSNDVIGHELTHGVTQFTAGLDYENEAGALNESVSDVFGSMFRQWQGQQSADKADWLIGKDILGPRALAKGYTCLRDMADPAAEHCLSPQPSHYRDYVPGSDPHEGSGIPNHAFYLAATKYGSHSWEAVGTVWYQALTSPKATKNMTFKAFAKLTRQIAAGRTGADSPQAAIDEAWTEVGL

>CORE_REP|Org31_Gene2150#

MCFIIPQDVLLRLADDDSVADDSRTALAATAASETAWRTLREAHTEATQAGLVARIDAFAGVAKALAKAPGTPVFDCKHTMSLPGVAVASPGSSTDASAKSAFTQTAAVAKFYKECFGRNSVDDEGMTLVSSVHYSVNYSNAFWNGSQMTYGDGDGEIFVDFTASNDVIGHELTHGVTQYTAGLLYKNEAGGLNESMSDVFGSMFRQWSAGQTVDQADWLIGKDIMGPRAVAKGFTCLRDMADPGARHCLAPQPSHYRDYVPGSDPHESSGIPNYAFYLAATKHGSYSWQGVGTVWYEALTSPKARPNMKMKAFANLTREISAANTATESVHKAIDDAWTAVGL

>CORE_REP|Org2_Gene4351#

MQQPRATSAAKRTHRARVIAATIAVTSLSGAGAYTLAATPADQAPATVRTANIHLVTTDAPPPPAPPATPAPGQPAPNPETPTTVTTPVPAPAPGQPAPPTPPPAPGAPADGGRVNNDQGGFSFVVPQGWVQSDARRLTYGSALLTNPAAPNGSILLGRLDLKLFAGAEPDNQKAARRLASDMGEFFMPYPGNRVNQEDQSFDVAGMSAASSYYEVKFDDAAKEPGQIWAAAVGKGKDRWFIVWLGTSASPVDKGVAKALTESIRPWTPPTPPTPSAAPPADPNQPAPPPADPNQPPPPPADPNQPPASPAPAAPAAPAAEPAAPGQPAPPAPPAAPPAPGVPV

>CORE_REP|Org14_Gene893#

MKLFSKMRGALARQSARRIAVAATAVAVLPGVAGIVGGTALTPVAGAFSRPGLPVEYLQVPSASMGREIKVQFQPGGAKAVYLLDGLRARDDFSGWDIETTAFEDYYQSGISMVMPVGGQSSFYTDWYNPAKGKDGVWTYKWETFTTQELPAYLAANKGISQTGNAVVGLSMGASAALTLSIYHPQLFVYAGALSGFLNPSDMKFQIGLAMGDAGGFSASDMWGPDSDPAWVRNDPFLNIQKIIDNGTRLWIYCGTGDSTDLDATRNGFENFTGGFLEGMAIGSNKKFVEAYTAAGGKNAHIEFPPGGIHNWTYWGQQLRAMKPDMVAYLQSH

>CORE_REP|Org10_Gene2417#

MKLFSQLRGKTARRLATVAAAAAVLPGFIGVAGGSAVANAFSRPGLPVEYLQVPSAAMNTSIKVQFQNGGAKSVYLLDGLRARDDFSGWDIETTAFEDYYQSGISVVMPVGGQSSWYTDWYQPAKGKDGVFTYKWETFLTQELPAFLANQGLSKTGNAVVGLSMGAASALNLANYHPQQFIYAGALSGFLHPADMKGQIGMAMGDAGGFNPQDMWGPDNDPAWVRNDPFLNIDRTVANGTRLWIYCGSGDATDLDATRNGFENFTGGFLEGMAIGSNKQYVDAYTAAGGKNAHVEFPPGGLHNWTYWGNQLKAMKSDMVGYLQSH

>CORE_REP|Org29_Gene4828#

MSVRVKARRRVLSALLAAFVMPVSMAAAMTINPATAHAFSREGLPVEYLDVYSNSMGRNIRVEFQGGGPKAVYLLDGLRAQDDFNGWDINTAAFEWFYQSGISVVMPVGGQSSFYTDWYSPSALNKQPYTYKWETFLTQELPAYLATNKQISATGNGVVGLSMSGGAALILAAFHPAQFRFAGSLSGFLNPSTIFMTNAIRVAMLDAGSYSVDNMWGPPWDPAWRRNDPTVQAQALVAAGTRLYIYCAPGGSTPIDDNTDAGVALSASSLESLAVAGNKAFQQAYTAAGGRNANFVFPASGNHSWPYWGQQLQALKGDLIATLNG

>CORE_REP|Org20_Gene1893#

MTDALHLILGDEELLVERAVTSVLHAVRGKAGADIPVNRLRAGQVDVAELAELLSPSLFADERVIVIEAAAEAGKDAVTLIEQAATDLPPGTFLLVQHSGGGRAKALATTLQNLGASVHNCARITKAAERADFVHKEFRRLGQKVDADVVAIVIDAVGSDIRELAAACSQLVSDTDGSVDGAAVRRYHSGRAEVSGFDIADKAVTGDIAGSTEALRWAMQRGVPHVLLADALAEAVHTIARVGPIKQNAYAAASELGMPPWRIEKAQKQARRWTRDAVAEAMRVVAALNADVKGVAADADYALESAVRRVAELVST

>CORE_REP|Org26_Gene2741#

MVNMDRRRMMAFSGLGMLAAAASMPQAWAQPSPLGPPNRPPAAPPTGKYVFVDEFDGPAGSAPDGSKWAISKARETMKDPTFWELPENVGQYRDDRKNVFLDGNSNLVIKAAKEGNTYYGGKIYSTTELGIGYTWEARIKFNCLTPGAWPAFWLGSDQDGEIDIVEWYGNGSWPSATTVHAKANGSEWKTHNISLDSGWHTWRTQWDDKGIRFWKDYTDGATPYFEVPANSLADWPFNNPGHKAFAVLNLAVAGSGGGDPRGGTYPAEMLVDWIRVW

>CORE_REP|Org12_Gene2307#

MRVGYLLIAAVCGVVLAGCGRGEVPSETAAPESADATTTSAPAVTADPGVQAACPKHGGRWDAGQGCVIDEATPQATQHLVVPVQWDSSFPELQQAVDGTVADIRANFRKSVERAGAPPEGKPWALQVSFETYQGKGAHPSDSVRFSISESLGGYHPGFAFRTLAFDRTSRQAITLDTLLIDPATALPKISALVRTDLRAQLGGVGAEFVDTGTVPEPGNFKDFSLDGDALLFSFEPYRVAAYAEGPMQSRIALSELRDVVKPEYLPA

>CORE_REP|Org31_Gene3580#

MAARRLYVAIIGTLGLCLSVVACGAQAPDPSGSRPSQHATAHQTGSAPADKRTDAYCAQNHDANCAAGTYLGPHAAPGAGAGYWDNNGNPVDGGPVGADGSTGNNVSQEYCARNEDPACPAGSYVDAKAIKNPDGSNSYVPCEGTVCTNPNHGGADEAGGWDSQGQPVNGGPKGADGSAGNNVSQEYCARNEDPACPAGSYVGPKAIKNPDGSNSYVPCEGTVCTNPNHGGGDNSGVPGNTDSPDPSGAGQDSPSGQ

>CORE_REP|Org30_Gene4808#

MNSIEHQLDRRQFLITAAASAGALALASCTNQTHEQTPPTKETGVYHLPNLDWDYSALEPHISGEINQLHHDKHHAAYVKGANDALEQLAQAREKGDNAAIVQLEKNLAFNLGGHANHSIWWKNLSPHGGDKPEGELGGAIDEQFGGFDKFRDQFSAAANGVQGSGWAWLGYDTLGKKLLTFQMYDQQSNVPFGTIPLLGLDVFEHAYYLQYKNVKADYIKAFWNVVNWPDVQARFAAAVSKTPGLIFP

>CORE_REP|Org14_Gene3036#

MQGLFLLRSAIAVTIAIALVLFMGVPRAAADDNPLGPNVGTAFLNALGLNPTGGQYDPTLPFAGPSQGADPAKIMNGVMGVGQTALGALGIGGNSASAGAGRPLVYGRAAVERVIQRGGTQLGVPYSWGGGTVRGPSGGVDYDSGKVGYDCSGFTMFSYAAAGVKLPKYSGDQYNAGQKVPVAQAKRGDLLFYGPGGSQHVVIYLGNGQMLEASGSAGKVTVSPVRTGGMTPYAVRIIAW

>CORE_REP|Org28_Gene2392#

MAEYTLPDLDYDYGALEPHISGQINELHHSKHHATYVKGVNDAVAKLEEAREKGDHAAIFLNEKNLAFHLGGHVNHSIWWKNLSPNGGDKPTGDLAAAIDDQFGSFDKFQAQFTAAANGLQGSGWAVLGYDSLGQKLLTFQLYDQQANVPLGIIPLLQVDMWEHAFYLQYKNVKADYVKAFWNVVNWADVQDRYTAATTKTSGLIFG

>CORE_REP|Org33_Gene1636#

MDTNLMKRAAGAVSIVAISAAVAVACSQQDKDAAKESVSSATSAASSAISAGGSAASSAASSASSAVSSVVAGAPSTVTVPGGGEVVLEPPIAEAYTKAGGEAKLGAPSGQPEKVGDGTVQAFAKGTIFSSPSTGAHLVQGEILKVYTAQGGAGGALGFPTADEEETAGGPDVAKGGWIGEFQKGTITWLNQGDGTFKETVTQK

>CORE_REP|Org21_Gene4576#

MRLLVAALAALALFITACSTGDDAVVRGGQFQFVSPGGKTDILYDPPQSRQTPGPISGPSLLDPTKTLSLSDFRGKVVVINVWGQWCGPCRSEFSALEDVYRATHAQGVEFLGINVRDNEITKAQDFVTDRKVPYPSIYDPSMRTLIAFGKKFPTGAIPATLVLDRQHRVAAVFLRELLAQDLRPVVERLAQESAKETAGEVK

>CORE_REP|Org30_Gene3896#

MARLNTGARWTIAGVVLVAALIAVMLSQQHDQQRRGPQGQQQVAARERRDAETPEALAELRRQAQLPSCPAANPDPGTPALAGITLECSGVRVPVGPVLAGRQVVLNLWAYWCGPCADELPAMAELQRRAGDKLTVITVHQDENEAAGLNRLAELHVRLPMIQDGARRIAAALKSPNVMPTTILIRADGSVAQVLPRSFTSADEIGAEVEQALGMRF

>CORE_REP|Org30_Gene1611#

MKKRLVSAGVIALAAALSGPAALAAAAPADEQQIRDIVAGEAAAIKSLDNAKLSTFFCDKFRGVVASKTADDQIPPLSQVAGYGPQMISAVASAAGVSGPTTQALTTAIQNNDDKAYRSSFHNAAREVLSGVTYNVSDVNVTAPTATADVTAQGHDVTANQQREFVQENGKWKDCTDPEKRAQTSGNPLLTSLLN

>CORE_REP|Org5_Gene3910#

MAALALALALSVGLSGCSGTDEKGEDSSAPSSRLLNFTAQTIDGADFAGSSLAGKKAVLWFWAPWCPTCQKEAPDLQKAATAHPDVTFVGVAAQDQVPAMRDFVTKYGLTFIQLADTDAKVWALYDVTHQPAFAFLGSEGKAEVVKSPLSGPELDKKIGQLH

>CORE_REP|Org20_Gene3810#

MAETKRLEVGDKAPAFTLLDADGKKVSLSSYKGRKVIIYFYPAAMTPGCTKQACDFRDSLAELNEAGLDVIGISPDKPEKLAKFRERDGVNFPLLSDPDKTTLTAYGAFGEKKLYGKIVEGVIRSTFVVDEKGNIEVAQYNVKAAHTTTKIQKILEQVMGTNRS

>CORE_REP|Org1_Gene665#

MSNLVWTRRSPFAEFDALVRQSFGPATSWPTPGFVPSADVVKDGDDALVRLDLPGVDVGKDVTVEVERGTLVVSGERRDERAEQTEGRTLREVRYGTFRRTFTLPTHVTGDAISASYDAGILTVKVAGAYANAAAQKIEITT

>CORE_REP|Org32_Gene579#

MQYRRVMSLRLRMVGTALLALALMGCTEPSPARATITQCNLSELPAEASGTVDLIHAGGPFPYPRNDGVVFQNRERVLPSEPRGYYHEYTVRTPGAKTRGTRRIITGGNPLNDPPHVYYTGDHYQSFCEVEGT

>CORE_REP|Org13_Gene1000#

MSRYMLSIVYAPGAVQPDEAALETIGADVQAVTRRMQDAGVWLFAAGLQPADTATMVTASAAGHTTTDGPYTETKEQLGGFSIIDVPTLADAQRWAAEVSRAVWCPIEVRGLDRGCGELD

>CORE_REP|Org29_Gene1754#

MKYVRKTLTTRAVLWAMAPALVAAPMALAGTASADPVNWDAIAACESGGNWGINTGNGYYGGLQFNLGTWRANGGSGSPHLASREEQIRVAENVLARQGIGAWPVCGRRG

>CORE_REP|Org16_Gene2585#

MPTYSYACADCGDKFDIVQSFTDDALTVCQKCSGKLRKLFNSVGIVFKGSGFYRTDSRSGSVDAATKSDAGTKSESKSSSDSSGSSSAGSSSSSSSSSSTATASAAAS

>CORE_REP|Org19_Gene2237#

MSTQSACARLLIFAFLGVTAAVGVDLMDGTNIPGGKEPAVTYSADPWDDEVEFLTGNDAMNIYTPDSRQINGQPQNIGGARNSNGIGKSCNNPGVRCR

>CORE_REP|Org23_Gene1578#

MNIKKVNIKRLIVTGVSAAAMGLATFAGAQGVANADPITPPPPPPIPQAPPIPDGNGVPPVPQAPPIPQIPAIPQP

>CORE_REP|Org21_Gene1303#

MIEIGSTFRRRGADGTWATFTIRVIRYSPFPYVEAEPVGGGPRVALSVRAAEGLSAAGG
