# Supplementary material for: Designing a multi-epitope vaccine against Mycobacteroides abscessus by pangenome-reverse vaccinology
Source: Sci Rep. 2021 May 27;11:11197. doi: 10.1038/s41598-021-90868-2 (PMC8159972; doi:10.1038/s41598-021-90868-2)
Supplement: Supplementary file 4 — Supplementary Information 4. [file 41598_2021_90868_MOESM4_ESM.docx]

**Designing a multi-epitope vaccine against *Mycobacteroides abscessus* by Pangenome-reverse vaccinology**

Hamza Arshad Dar^1#^, Saba Ismail^1#^, Yasir Waheed^1*^, Sajjad Ahmad^1^, Zubia Jamil^1^, Hafsa Aziz^2^, Helal F. Hetta^3,4^, Khalid Muhammad^5*^.

1. Foundation University Medical College, Foundation University Islamabad, DHA-I, Islamabad 44000, Pakistan
2. Nuclear Medicine, Oncology, and Radiotherapy Institute, Islamabad 44000, Pakistan.
3. Department of Internal Medicine, University of Cincinnati College of Medicine, 231 Albert Sabin Way, Cincinnati, OH 45267-0595, USA.
4. Department of Medical Microbiology and Immunology, Faculty of Medicine, Assiut University, Assiut 71515, Egypt.
5. Department of Biology, College of Science, United Arab Emirates University, Al Ain, 15551, United Arab Emirates.

*Correspondence: [yasir.waheed@fui.edu.pk](mailto:yasir.waheed@fui.edu.pk)*,* [*k.muhammad@uaeu.ac.ae*](mailto:k.muhammad@uaeu.ac.ae)

>CORE_REPOrg29_Gene3335

MANKWDIEALRGEGLQAIANSQNYVTAAIRGNGKSPVTITNPDLTANERQLFDWYDMDAG

MDLNTLGGDLELFKNATATMKAAAERQHGQLQRLIGLWEGKGSESANDFLKTHNSTADAV

TDEFGKVSTGLDGLRNALWNIVDLKKQASTMVDGLVTDRTHFDSAVATYKTGMGDKSQAD

ETNATMIGPHVKNNIEGQLLPAFKKAWSAGGGAYDTLINGLKQELPPDFKLPPGVFGPDY

DTTDEPAKTTKGKGKQDDKDGGETSGESGESGNSGVNSGAGGGTASGGMQGTATPASATG

NAGGQLSGAGQQQGAGQGQQGMDPSQMLSGMTGALTGALSSIGQAASGIVSAITEGISSI

PFDQMGQGLGDDQFDGRADEAADKKDEAAADGKKDPDAKMAAAKDAAIEEARADSGATFA

TDGKPAPGIQLAGAGGLEATPTAAPGQTTPGAPLGATPTGTIPPAAGGLSAAQPAGSSAS

LTPHPVQAQPPTVPQHPEPQSAAARQPSPLPSVGPTDASAQPQEAKTEAGETPCEIAADE

LPKAGR

>CORE_REPOrg31_Gene3714

MKRTVNDRFAVRGKRTVATALMVPPLMVAGLMMFPTTVAVTSAEPNDMASLITQLADTNQ

QIEQLTADVQTQQESINKGLVDLQAARDNAASAAAQVAEGQRAVDAANGAIEEAQGKFDR

MAAATYMAGPSTSYLTATNPDDVVRLASVTKSVEASSQTVMDNLRRARTEQVNKQSQARA

IQEKADQAAADAQQQQDDLVSAMKDVQKKLEAQRGVAADLTAKKKSAEAQLAAARGPAYA

ASTATARVINPSAAIAGNGNEWTEGPAPVSSGGQWDTTLPMIASANVPTDPTQTINMVLG

IGNTAANVGQSAVCGVIGIFCPKAAPAAAASGEGGEYLPKVYGRENVERVIARAGSALGT

PYSWGGGSYNGPTRGIDSGAGTVGYDCSGLMMYGFAAVGIRLRHYTGYQYNSGRKVPSAQ

MKRGDMIFYGPNASQHVALYLGNGQMLEAPNTGDVVKVSPVRTSGMTPYVTRMIEW

>CORE_REPOrg10_Gene1299

MNALTKLNATRSPLLRALVAALLVVIGAGGGYAIAAHKTLTLNVDGNAMTVTTVKSRVSE

VLADYGYALSDRDDVAPAKHDSVRDGDTIVLKRSRPLDISVDGQDTQQVWTTASTVNDAL

SQLSMTDTAPTAATRGSRLPLEGMSLAVVSAKTVQLNDGGVISTPRIAAPTVGALLEATG

NPLQQFDTVDPAPSTPVTADMPITVTRIRVSKVTEQAPLAPTPQKIEDPEMNMSRSVVQD

PGAPGTQDIVYSVLSVNGRETGRIPVSNNVLTPARDSVLRVGAKPGTEVPAVTRGSAWDA

LAQCEAGGNWAINTGNGFYGGVQFDYGTWLAHGGGKYAPRADLATREEQIAIAEKTLSAQ

GWGAWPVCSARVGAR

>CORE_REPOrg24_Gene1824

MCQRFIIPPAVLSRLAEDGDIAEDSRAALSATAACELSWRTLRNAHTRATQASLLTSRAA

ALGAATTGLAKIPETPVFDCRQTTSLPGVAVADPATSKDATAQRAFDETAAVAQFYRNCF

GRNSVDNAGMTLVSSIHYGVKYSNAFWNGSQMAYGDGDGQIFLDFTKSNDVIGHELTHGV

TQFTAGLDYENEAGALNESVSDVFGSMFRQWQGQQSADKADWLIGKDILGPRALAKGYTC

LRDMADPAAEHCLSPQPSHYRDYVPGSDPHEGSGIPNHAFYLAATKYGSHSWEAVGTVWY

QALTSPKATKNMTFKAFAKLTRQIAAGRTGADSPQAAIDEAWTEVGL

>CORE_REPOrg31_Gene2150

MCFIIPQDVLLRLADDDSVADDSRTALAATAASETAWRTLREAHTEATQAGLVARIDAFA

GVAKALAKAPGTPVFDCKHTMSLPGVAVASPGSSTDASAKSAFTQTAAVAKFYKECFGRN

SVDDEGMTLVSSVHYSVNYSNAFWNGSQMTYGDGDGEIFVDFTASNDVIGHELTHGVTQY

TAGLLYKNEAGGLNESMSDVFGSMFRQWSAGQTVDQADWLIGKDIMGPRAVAKGFTCLRD

MADPGARHCLAPQPSHYRDYVPGSDPHESSGIPNYAFYLAATKHGSYSWQGVGTVWYEAL

TSPKARPNMKMKAFANLTREISAANTATESVHKAIDDAWTAVGL

>CORE_REPOrg2_Gene4351

MQQPRATSAAKRTHRARVIAATIAVTSLSGAGAYTLAATPADQAPATVRTANIHLVTTDA

PPPPAPPATPAPGQPAPNPETPTTVTTPVPAPAPGQPAPPTPPPAPGAPADGGRVNNDQG

GFSFVVPQGWVQSDARRLTYGSALLTNPAAPNGSILLGRLDLKLFAGAEPDNQKAARRLA

SDMGEFFMPYPGNRVNQEDQSFDVAGMSAASSYYEVKFDDAAKEPGQIWAAAVGKGKDRW

FIVWLGTSASPVDKGVAKALTESIRPWTPPTPPTPSAAPPADPNQPAPPPADPNQPPPPP

ADPNQPPASPAPAAPAAPAAEPAAPGQPAPPAPPAAPPAPGVPV

>CORE_REPOrg14_Gene893

MKLFSKMRGALARQSARRIAVAATAVAVLPGVAGIVGGTALTPVAGAFSRPGLPVEYLQV

PSASMGREIKVQFQPGGAKAVYLLDGLRARDDFSGWDIETTAFEDYYQSGISMVMPVGGQ

SSFYTDWYNPAKGKDGVWTYKWETFTTQELPAYLAANKGISQTGNAVVGLSMGASAALTL

SIYHPQLFVYAGALSGFLNPSDMKFQIGLAMGDAGGFSASDMWGPDSDPAWVRNDPFLNI

QKIIDNGTRLWIYCGTGDSTDLDATRNGFENFTGGFLEGMAIGSNKKFVEAYTAAGGKNA

HIEFPPGGIHNWTYWGQQLRAMKPDMVAYLQSH

>CORE_REPOrg10_Gene2417

MKLFSQLRGKTARRLATVAAAAAVLPGFIGVAGGSAVANAFSRPGLPVEYLQVPSAAMNT

SIKVQFQNGGAKSVYLLDGLRARDDFSGWDIETTAFEDYYQSGISVVMPVGGQSSWYTDW

YQPAKGKDGVFTYKWETFLTQELPAFLANQGLSKTGNAVVGLSMGAASALNLANYHPQQF

IYAGALSGFLHPADMKGQIGMAMGDAGGFNPQDMWGPDNDPAWVRNDPFLNIDRTVANGT

RLWIYCGSGDATDLDATRNGFENFTGGFLEGMAIGSNKQYVDAYTAAGGKNAHVEFPPGG

LHNWTYWGNQLKAMKSDMVGYLQSH

>CORE_REPOrg29_Gene4828

MSVRVKARRRVLSALLAAFVMPVSMAAAMTINPATAHAFSREGLPVEYLDVYSNSMGRNI

RVEFQGGGPKAVYLLDGLRAQDDFNGWDINTAAFEWFYQSGISVVMPVGGQSSFYTDWYS

PSALNKQPYTYKWETFLTQELPAYLATNKQISATGNGVVGLSMSGGAALILAAFHPAQFR

FAGSLSGFLNPSTIFMTNAIRVAMLDAGSYSVDNMWGPPWDPAWRRNDPTVQAQALVAAG

TRLYIYCAPGGSTPIDDNTDAGVALSASSLESLAVAGNKAFQQAYTAAGGRNANFVFPAS

GNHSWPYWGQQLQALKGDLIATLNG

>CORE_REPOrg26_Gene2741

MVNMDRRRMMAFSGLGMLAAAASMPQAWAQPSPLGPPNRPPAAPPTGKYVFVDEFDGPAG

SAPDGSKWAISKARETMKDPTFWELPENVGQYRDDRKNVFLDGNSNLVIKAAKEGNTYYG

GKIYSTTELGIGYTWEARIKFNCLTPGAWPAFWLGSDQDGEIDIVEWYGNGSWPSATTVH

AKANGSEWKTHNISLDSGWHTWRTQWDDKGIRFWKDYTDGATPYFEVPANSLADWPFNNP

GHKAFAVLNLAVAGSGGGDPRGGTYPAEMLVDWIRVW

>CORE_REPOrg31_Gene3580

MAARRLYVAIIGTLGLCLSVVACGAQAPDPSGSRPSQHATAHQTGSAPADKRTDAYCAQN

HDANCAAGTYLGPHAAPGAGAGYWDNNGNPVDGGPVGADGSTGNNVSQEYCARNEDPACP

AGSYVDAKAIKNPDGSNSYVPCEGTVCTNPNHGGADEAGGWDSQGQPVNGGPKGADGSAG

NNVSQEYCARNEDPACPAGSYVGPKAIKNPDGSNSYVPCEGTVCTNPNHGGGDNSGVPGN

TDSPDPSGAGQDSPSGQ

>CORE_REPOrg14_Gene3036

MQGLFLLRSAIAVTIAIALVLFMGVPRAAADDNPLGPNVGTAFLNALGLNPTGGQYDPTL

PFAGPSQGADPAKIMNGVMGVGQTALGALGIGGNSASAGAGRPLVYGRAAVERVIQRGGT

QLGVPYSWGGGTVRGPSGGVDYDSGKVGYDCSGFTMFSYAAAGVKLPKYSGDQYNAGQKV

PVAQAKRGDLLFYGPGGSQHVVIYLGNGQMLEASGSAGKVTVSPVRTGGMTPYAVRIIAW

>CORE_REPOrg33_Gene1636

MDTNLMKRAAGAVSIVAISAAVAVACSQQDKDAAKESVSSATSAASSAISAGGSAASSAA

SSASSAVSSVVAGAPSTVTVPGGGEVVLEPPIAEAYTKAGGEAKLGAPSGQPEKVGDGTV

QAFAKGTIFSSPSTGAHLVQGEILKVYTAQGGAGGALGFPTADEEETAGGPDVAKGGWIG

EFQKGTITWLNQGDGTFKETVTQK

>CORE_REPOrg30_Gene1611

MKKRLVSAGVIALAAALSGPAALAAAAPADEQQIRDIVAGEAAAIKSLDNAKLSTFFCDK

FRGVVASKTADDQIPPLSQVAGYGPQMISAVASAAGVSGPTTQALTTAIQNNDDKAYRSS

FHNAAREVLSGVTYNVSDVNVTAPTATADVTAQGHDVTANQQREFVQENGKWKDCTDPEK

RAQTSGNPLLTSLLN

>CORE_REPOrg5_Gene3910

MAALALALALSVGLSGCSGTDEKGEDSSAPSSRLLNFTAQTIDGADFAGSSLAGKKAVLW

FWAPWCPTCQKEAPDLQKAATAHPDVTFVGVAAQDQVPAMRDFVTKYGLTFIQLADTDAK

VWALYDVTHQPAFAFLGSEGKAEVVKSPLSGPELDKKIGQLH

>CORE_REPOrg29_Gene1754

MKYVRKTLTTRAVLWAMAPALVAAPMALAGTASADPVNWDAIAACESGGNWGINTGNGYY

GGLQFNLGTWRANGGSGSPHLASREEQIRVAENVLARQGIGAWPVCGRRG

>CORE_REPOrg16_Gene2585

MPTYSYACADCGDKFDIVQSFTDDALTVCQKCSGKLRKLFNSVGIVFKGSGFYRTDSRSG

SVDAATKSDAGTKSESKSSSDSSGSSSAGSSSSSSSSSSTATASAAAS

>CORE_REPOrg19_Gene2237

MSTQSACARLLIFAFLGVTAAVGVDLMDGTNIPGGKEPAVTYSADPWDDEVEFLTGNDAM

NIYTPDSRQINGQPQNIGGARNSNGIGKSCNNPGVRCR

>CORE_REPOrg23_Gene1578

MNIKKVNIKRLIVTGVSAAAMGLATFAGAQGVANADPITPPPPPPIPQAPPIPDGNGVPP

VPQAPPIPQIPAIPQP
