# Supplementary material for: Designing a multi-epitope vaccine against Mycobacteroides abscessus by pangenome-reverse vaccinology
Source: Sci Rep. 2021 May 27;11:11197. doi: 10.1038/s41598-021-90868-2 (PMC8159972; doi:10.1038/s41598-021-90868-2)
Supplement: Supplementary file 6 — Supplementary Information 6. [file 41598_2021_90868_MOESM6_ESM.docx]

**Designing a multi-epitope vaccine against *Mycobacteroides abscessus* by Pangenome-reverse vaccinology**

Hamza Arshad Dar^1#^, Saba Ismail^1#^, Yasir Waheed^1*^, Sajjad Ahmad^1^, Zubia Jamil^1^, Hafsa Aziz^2^, Helal F. Hetta^3,4^, Khalid Muhammad^5*^.

1. Foundation University Medical College, Foundation University Islamabad, DHA-I, Islamabad 44000, Pakistan
2. Nuclear Medicine, Oncology, and Radiotherapy Institute, Islamabad 44000, Pakistan.
3. Department of Internal Medicine, University of Cincinnati College of Medicine, 231 Albert Sabin Way, Cincinnati, OH 45267-0595, USA.
4. Department of Medical Microbiology and Immunology, Faculty of Medicine, Assiut University, Assiut 71515, Egypt.
5. Department of Biology, College of Science, United Arab Emirates University, Al Ain, 15551, United Arab Emirates.

*Correspondence: [yasir.waheed@fui.edu.pk](mailto:yasir.waheed@fui.edu.pk)*,* [*k.muhammad@uaeu.ac.ae*](mailto:k.muhammad@uaeu.ac.ae)

>CORE_REPOrg29_Gene3335

MANKWDIEALRGEGLQAIANSQNYVTAAIRGNGKSPVTITNPDLTANERQLFDWYDMDAG

MDLNTLGGDLELFKNATATMKAAAERQHGQLQRLIGLWEGKGSESANDFLKTHNSTADAV

TDEFGKVSTGLDGLRNALWNIVDLKKQASTMVDGLVTDRTHFDSAVATYKTGMGDKSQAD

ETNATMIGPHVKNNIEGQLLPAFKKAWSAGGGAYDTLINGLKQELPPDFKLPPGVFGPDY

DTTDEPAKTTKGKGKQDDKDGGETSGESGESGNSGVNSGAGGGTASGGMQGTATPASATG

NAGGQLSGAGQQQGAGQGQQGMDPSQMLSGMTGALTGALSSIGQAASGIVSAITEGISSI

PFDQMGQGLGDDQFDGRADEAADKKDEAAADGKKDPDAKMAAAKDAAIEEARADSGATFA

TDGKPAPGIQLAGAGGLEATPTAAPGQTTPGAPLGATPTGTIPPAAGGLSAAQPAGSSAS

LTPHPVQAQPPTVPQHPEPQSAAARQPSPLPSVGPTDASAQPQEAKTEAGETPCEIAADE

LPKAGR

>CORE_REPOrg31_Gene3714

MKRTVNDRFAVRGKRTVATALMVPPLMVAGLMMFPTTVAVTSAEPNDMASLITQLADTNQ

QIEQLTADVQTQQESINKGLVDLQAARDNAASAAAQVAEGQRAVDAANGAIEEAQGKFDR

MAAATYMAGPSTSYLTATNPDDVVRLASVTKSVEASSQTVMDNLRRARTEQVNKQSQARA

IQEKADQAAADAQQQQDDLVSAMKDVQKKLEAQRGVAADLTAKKKSAEAQLAAARGPAYA

ASTATARVINPSAAIAGNGNEWTEGPAPVSSGGQWDTTLPMIASANVPTDPTQTINMVLG

IGNTAANVGQSAVCGVIGIFCPKAAPAAAASGEGGEYLPKVYGRENVERVIARAGSALGT

PYSWGGGSYNGPTRGIDSGAGTVGYDCSGLMMYGFAAVGIRLRHYTGYQYNSGRKVPSAQ

MKRGDMIFYGPNASQHVALYLGNGQMLEAPNTGDVVKVSPVRTSGMTPYVTRMIEW

>CORE_REPOrg29_Gene1754

MKYVRKTLTTRAVLWAMAPALVAAPMALAGTASADPVNWDAIAACESGGNWGINTGNGYY

GGLQFNLGTWRANGGSGSPHLASREEQIRVAENVLARQGIGAWPVCGRRG

>CORE_REPOrg19_Gene2237

MSTQSACARLLIFAFLGVTAAVGVDLMDGTNIPGGKEPAVTYSADPWDDEVEFLTGNDAM

NIYTPDSRQINGQPQNIGGARNSNGIGKSCNNPGVRCR
