# Supplementary material for: Designing a multi-epitope vaccine against Mycobacteroides abscessus by pangenome-reverse vaccinology
Source: Sci Rep. 2021 May 27;11:11197. doi: 10.1038/s41598-021-90868-2 (PMC8159972; doi:10.1038/s41598-021-90868-2)
Supplement: Supplementary file 13 — Supplementary Information 13. [file 41598_2021_90868_MOESM13_ESM.docx]

**Designing a multi-epitope vaccine against *Mycobacteroides abscessus* by Pangenome-reverse vaccinology**

Hamza Arshad Dar^1#^, Saba Ismail^1#^, Yasir Waheed^1*^, Sajjad Ahmad^1^, Zubia Jamil^1^, Hafsa Aziz^2^, Helal F. Hetta^3,4^, Khalid Muhammad^5*^.

1. Foundation University Medical College, Foundation University Islamabad, DHA-I, Islamabad 44000, Pakistan
2. Nuclear Medicine, Oncology, and Radiotherapy Institute, Islamabad 44000, Pakistan.
3. Department of Internal Medicine, University of Cincinnati College of Medicine, 231 Albert Sabin Way, Cincinnati, OH 45267-0595, USA.
4. Department of Medical Microbiology and Immunology, Faculty of Medicine, Assiut University, Assiut 71515, Egypt.
5. Department of Biology, College of Science, United Arab Emirates University, Al Ain, 15551, United Arab Emirates.

*Correspondence: [yasir.waheed@fui.edu.pk](mailto:yasir.waheed@fui.edu.pk)*,* [*k.muhammad@uaeu.ac.ae*](mailto:k.muhammad@uaeu.ac.ae)

# Multi-epitope vaccine CPORT Results

Predicted residues (active residues in HADDOCK):

13, 14, 15, 26, 27, 28, 29, 30, 32, 33, 34, 36, 42, 77, 89,

98, 118, 119, 130, 137, 144, 145, 146, 147, 158, 162, 164, 165, 168, 171,

191, 192, 193, 200, 201, 202, 204, 205, 206, 208, 209, 210, 211, 213, 214,

215, 216, 217, 218, 219, 220, 221, 222, 223, 224, 225, 226

Surrounding residues (passive residues in HADDOCK):

8, 11, 12, 16, 17, 21, 23, 24, 31, 35, 37, 38, 40, 44, 45,

46, 47, 48, 52, 53, 57, 58, 62, 71, 74, 75, 78, 79, 80, 90,

91, 92, 93, 94, 95, 97, 115, 116, 120, 121, 122, 125, 126, 127, 142,

148, 152, 153, 154, 155, 156, 157, 159, 161, 166, 167, 169, 170, 172, 173,

174, 175, 176, 177, 178, 179, 181, 182, 183, 185, 186, 187, 188, 190, 194,

195, 196, 197, 198, 199

# TLR2 CPORT Results

Predicted residues (active residues in HADDOCK):

27, 29, 30, 31, 32, 33, 35, 48, 63, 65, 66, 85, 86, 87, 89,

109, 111, 133, 263, 291, 292, 294, 321, 322, 323, 324, 325, 326, 327, 329,

330, 347, 348, 349, 350, 352, 353, 370, 371, 373, 376, 379, 398, 516, 537,

538, 539, 540, 541, 543, 544, 547, 550, 570, 571, 572, 573, 574, 575

Surrounding residues (passive residues in HADDOCK):

28, 34, 37, 38, 39, 40, 41, 42, 44, 45, 46, 47, 49, 51, 53,

55, 56, 57, 60, 61, 68, 70, 71, 74, 82, 84, 90, 91, 92, 113,

114, 135, 159, 160, 262, 264, 266, 288, 290, 295, 296, 297, 298, 317, 318,

319, 320, 328, 331, 332, 333, 334, 343, 344, 345, 346, 351, 354, 357, 358,

369, 375, 378, 382, 383, 384, 395, 396, 397, 400, 422, 424, 512, 513, 514,

515, 517, 518, 519, 520, 521, 534, 536, 551, 552, 553, 554, 556, 558, 559,

561, 562, 563, 564, 565, 567, 568
