# Supplementary material for: Designing a multi-epitope vaccine against Mycobacteroides abscessus by pangenome-reverse vaccinology
Source: Sci Rep. 2021 May 27;11:11197. doi: 10.1038/s41598-021-90868-2 (PMC8159972; doi:10.1038/s41598-021-90868-2)
Supplement: Supplementary file 14 — Supplementary Information 14. [file 41598_2021_90868_MOESM14_ESM.docx]

Supplementary file 1: The information of fully sequenced bacterial genomes such as strain name, accession number, as well as genome statistics.

Supplementary file 2: The core proteins having subcellular localization restricted/confined to the extracellular and cell wall regions.

Supplementary file 3: Homology analysis to retain non-homologous proteins.

Supplementary file 4: Proteins showing putative adhesion properties.

Supplementary file 5: Antigenic protein sequences.

Supplementary file 6: Potential protein targets obtained after physicochemical analysis.

Supplementary file 7: Validation of subcellular localization.

Supplementary file 8: VaxiTop predicted HLA I T-cell epitopes within the four core vaccine targets.

Supplementary file 9: The predicted HLA I T-cell epitopes showing positive immunogenicity score.

Supplementary file 10: The list of preliminary HLA II T-cell epitopes predicted within four vaccine targets.

Supplementary file 11: Discontinuous B-cell epitope prediction of vaccine by Ellipro server.

Supplementary file 12: Discontinuous B-cell epitope prediction of vaccine by DISCOTOPE server.

Supplementary file 13: CPORT results showing active as well as passive residues of Multiepitope and TLR2.
